# Impact of Single Nucleotide Polymorphisms of Base Excision Repair Genes on DNA Damage and Efficiency of DNA Repair in Recurrent Depression Disorder

**DOI:** 10.1007/s12035-016-9971-6

**Published:** 2016-06-21

**Authors:** Piotr Czarny, Dominik Kwiatkowski, Monika Toma, Joanna Kubiak, Agnieszka Sliwinska, Monika Talarowska, Janusz Szemraj, Michael Maes, Piotr Galecki, Tomasz Sliwinski

**Affiliations:** 1 Department of Molecular Genetics, University of Lodz, 141/143 Pomorska Street, 90-236 Lodz, Poland; 2 Department of Medical Biochemistry, Medical University of Lodz, Lodz, Poland; 3 Department of Internal Disease, Diabetology and Clinical Pharmacology, Medical University of Lodz, Lodz, Poland; 4 Department of Adult Psychiatry, Medical University of Lodz, Lodz, Poland; 5 IMPACT Strategic Research Centre, Barwon Health, Deakin University School of Medicine, Geelong, Victoria Australia; 6 Department of Psychiatry, Chulalongkorn University, Bangkok, Thailand; 7 Health Sciences Graduate Program, Health Sciences Center, State University of Londrina, Londrina, Brazil

**Keywords:** Recurrent depression disorder, DNA damage, DNA repair, Oxidative stress, Base excision repair, Single nucleotide polymorphism

## Abstract

Elevated level of DNA damage was observed in patients with depression. Furthermore, single nucleotide polymorphisms (SNPs) of base excision repair (BER) genes may modulate the risk of this disease. Therefore, the aim of this study was to delineate the association between DNA damage, DNA repair, the presence of polymorphic variants of BER genes, and occurrence of depression. The study was conducted on peripheral blood mononuclear cells of 43 patients diagnosed with depression and 59 controls without mental disorders. Comet assay was used to assess endogenous (oxidative) DNA damage and efficiency of DNA damage repair (DRE). TaqMan probes were employed to genotype 12 SNPs of BER genes. Endogenous DNA damage was higher in the patients than in the controls, but none of the SNPs affected its levels. DRE was significantly higher in the controls and was modulated by BER SNPs, particularly by c.977C>G–*hOGG1*, c.972G>C–*MUTYH*, c.2285T>C–*PARP1*, c.580C>T–*XRCC1*, c.1196A>G–*XRCC1*, c.444T>G–*APEX1*, c.-468T>G–*APEX1*, or c.*50C>T–*LIG3*. Our study suggests that both oxidative stress and disorders in DNA damage repair mechanisms contribute to elevated levels of DNA lesions observed in depression. Lower DRE can be partly attributed to the presence of specific SNP variants.

**Electronic supplementary material:**

The online version of this article (doi:10.1007/s12035-016-9971-6) contains supplementary material, which is available to authorized users.

## Introduction

Even though depression disorder (including the recurrent type—rDD) is common, its pathogenesis still remains elusive. Activation of immune-inflammatory pathways plays a key role in the onset of depression [1, 2]. It is indicated by high levels of pro-inflammatory cytokines and elevated expression of the NOD-like receptor family, pyrin domain containing 3 (NLRP3), a component of the inflammasome which releases pro-inflammatory cytokines [3–6]. NLRP3 is involved in DNA damage response (DDR) since its knockout increases the effectiveness of double-strand break repair and base excision repair (BER) [7]. The activated immune-inflammatory pathways present in depression often coexist with increased oxidative stress, as indicated by elevated levels of lipid peroxidation and production of reactive oxygen species (ROS) in patients affected by the disease [6, 8]. Oxidative damage to DNA is one of the consequences of oxidative stress in depression and is indicated by increased 8-oxoguanine (8-oxoG) in the urine, serum, and peripheral blood mononuclear cells (PBMCs) of depressed patients [9–14]. However, the urinary levels of 8-oxoG in patients with milder, non-clinical depression did not differ from the healthy controls [15]. Our previous study utilizing comet assay technique on the PBMCs of patients diagnosed with clinical depression confirmed the presence of not only oxidatively modified purines and pyrimidines but also other types of DNA damage, like DNA strand breaks [16]. Furthermore, we also noted that increased DNA damage in patients with depression might be caused not only by the disease itself but also by the impairments of oxidative DNA damage repair, since we observed that the patients’ cells repaired DNA damage induced by hydrogen peroxide (H_2_O_2_) more slowly than the controls’ cells [17]. Since it was shown that single nucleotide polymorphism (SNP) variants of genes involved in DDR, particularly BER, may negatively affect the process, we genotyped the SNPs of genes involved in this repair pathway [16–18]. We showed that some of these polymorphic variants increased the risk of rDD while others decreased this risk [16].

Therefore, in the present study, we delineate whether SNPs of genes involved in BER may affect DNA damage repair. We have genotyped 12 SNPs located in either coding or regulatory regions of BER genes and estimated the level of endogenous DNA damage as well as the efficiency of DNA damage repair (DRE) in PBMCs of patients with diagnosed rDD and healthy controls.

## Material and Methods

### Patients

Participants of this study consisted of 43 patients with rDD (mean age, 49.3 ± 10.2 years) hospitalized at the Department of Adult Psychiatry of the Medical University of Lodz (Poland) and 59 healthy controls (mean age, 51.2 ± 13.3 years) randomly selected without replacement sampling. Inclusion criteria and diagnosis were based on those outlined in ICD-10 (F32.0-7.32.2 and F33.0-F33.8) [20]. Standardized Composite International Diagnostic Interview (CIDI) was used to obtain a medical history for all cases [21]. Cases with axis I and axis II disorders other than depression, inflammatory, or autoimmune disorders were excluded as well as those with severe and chronic somatic diseases or worsening of symptoms and injuries of the central nervous system. Blood samples were taken from the rDD patients before they were subjected to an antidepressant therapy with drugs from the selective serotonin reuptake inhibitor group. Detailed information about patients are presented in Table 1. Respondents without cases of mental illness in their medical history were randomly selected to form the control group. According to the protocol approved by the Bioethics Committee of the Medical University of Lodz (no. RNN/70/14/KE), an informed written consent was obtained from each participant.

**Table 1:** Course of depression in the rDD group

Variable	rDD (*N* = 43), *M* (SD)
HDRS	23.74 (5.74)
No. of depression episodes	6.92 (5.81)
Disease duration	4.63 (3.21)

*rDD* recurrent depressive disorder, *HDRS* Hamilton Depression Rating Scale at the onset of therapy, *M* mean, *SD* standard deviation

### Blood Collection

Ten milliliters of venous blood was taken from the participants after period of fasting. Blood samples were stored at 4 °C and the isolation of PBMCs was done within 4 h after the collection. Additionally, blood from depressed patients was obtained before the pharmacological treatment started. An aliquot of 500 μl of blood was frozen and stored at −20 °C until the isolation of DNA.

### DNA Isolation

DNA was isolated from venous blood using Blood Mini Kit (A&A Biotechnology, Gdynia, Poland). Its purity was controlled by acquiring the absorbance ratio at 260 and 280 nm.

### Selection of Single Nucleotide Polymorphism and Genotyping

Selection of the SNPs was performed using the public domain of the National Center for Biotechnology Information, the Single Nucleotide Polymorphisms database at http://www.ncbi.nlm.nih.gov/snp (Bethesda, MD, USA). The following criteria were used to choose the SNPs: a minor allele frequency higher than 0.05 in a European population (submitter population ID: HapMap-CEU); localization in coding region causing non-synonymous substitution or in regulatory regions; and presence in the literature concerning diseases other than depression in pathogenesis in which oxidative stress and increased DNA damage play a major role.

TaqMan® SNP Genotyping Assay with TaqMan Fast Universal PCR Master mix (Life Technologies, Carlsbad, CA, USA) were used to genotype the studied SNPs. Reactions were performed in conditions recommended by the manufacturer in a Bio-Rad CFX96 thermal cycler with Real-Time PCR Detection System (Bio-Rad Laboratories Inc., Hercules, CA, USA). Results were analyzed in CFX Manager Software (Bio-Rad Laboratories Inc.).

### Peripheral Blood Mononuclear Cell Isolation and Treatment

Isolation of PBMCs was performed by isopycnic centrifugation (30°min, 400×*g*, 4 °C) of blood in Histopaque-1077 (Sigma-Aldrich, St. Louis, MO, USA). The obtained cells were then immediately counted in Bürcker chamber and used in the experiments. The level of DNA damage detected in untreated PBMCs was considered as endogenous DNA damage. To assess the efficacy of DDR, firstly, PBMCs were exposed to 20 μM H_2_O_2_ for 10 min on ice (POCH S.A., Gliwice, Poland). Then, PBMCs were washed and suspended in fresh RPMI-1640 medium for 120 min at 37 °C and the level of DNA damage was measured at the beginning and after 120 min of repair incubation.

### Comet Assay

DNA damage in PBMCs was estimated by the alkaline version (pH > 13) of the comet assay according to the procedure described by Singh et al. with later modifications [22–24]. Since this technique recognizes only DNA strand breaks and alkali labile sites, two glycosylases—Nth or human 8-oxoguanine DNA glycosylase (endonuclease III and hOOG1, respectively; New England Biolabs, Ipswich, MA, USA)—were used to detect oxidative DNA damage according to the procedure described earlier [25]. hOOG1 recognizes and removes 8-oxoadenine when paired with cytosine and 7,8-dihydro-8-oxoguanine (8-oxoguanine) when paired with cytosine, methyl-fapy-guanine, and foramidopyrimidine (fapy)-guanine [26, 27]. Nth recognizes and removes urea, thymine glycol, 5,6-dihydroxythymine, uracil glycol, 5-hydroxy-5-methylhydanton, 6-hydroxy-5,6-dihdrothimine, and methyltartronylurea [28, 29].

Each sample containing 5 × 10^5^ PBMCs was centrifuged (250×*g*, 15 min, 4 °C), suspended in 1.13 % agarose type XI (Sigma-Aldrich), cast on a microscope slide pre-coated with 0.5% low electroendosmosis agarose type I (Sigma-Aldrich), covered with a cover glass, and gelled on a cold plate for 10 min. Then, the cover glass was removed and the slide was incubated overnight in a lysis solution (2.5 mM NaCl, 100 mM Tris, 10 mM EDTA, 1 % Triton X-100, pH 10) at 4 °C. After lysis, slides were left for 20 min in buffer containing 300 mM NaOH and 1 mM EDTA, allowing the unwinding of DNA. Lastly, the 20-min electrophoresis was conducted at an electric field strength of 0.73 V/cm (300 mA) in a buffer of pH > 13 (300 mM NaOH and 1 mM EDTA).

The incubation with glycosylases was performed after lysis. These samples were washed with enzyme buffer (0.1 M KCl, 0.5 mM EDTA, 40 mM HEPES, 0.2 mg/ml bovine serum albumin, pH 8) and treated for 1 h with 1 U of OGG1/Nth, or with the buffer alone as a control at 37 °C. After incubation completion, unwinding and electrophoresis were conducted as described previously.

When electrophoresis ended, slides were washed twice with deionized water and left to dry out. For analysis, samples were stained for at least 60 min with 1 μg/ml DAPI, and then the comet pictures were captured in an Eclipse fluorescence microscope (Nikon, Tokyo, Japan) with a COHU 4910 video camera (Cohu, San Diego, CA, USA) and measured with Lucia-Comet image analysis system (Laboratory Imaging, Praha, Czech Republic). For each sample, 50 comets were analyzed; DNA damage levels are presented as a mean of the percentage of DNA in the tail of the comets. Results for samples digested with the DNA repair enzymes were normalized by subtracting the level of DNA damage evoked by enzyme buffer only.

### Statistical Analysis

Statistical analysis of the gathered data was performed in Statistica 12 (Statsoft, Tulsa, OK, USA) and SigmaPlot 11.0 (Systat Software Inc., San Jose, CA, USA). The association between case/control and each polymorphism was calculated using an unconditional multiple logistic regression model, and the results are shown as odds ratios (ORs) with 95 % confidence interval (95% CI). Data presenting the results from the comet assay analysis are shown as the mean ± SEM from two separate experiments. Normality of distribution of the data was examined by the Kolmogorov–Smirnov test and equality of variances by *F* test. Significance of the difference between the studied values was determined by Mann–Whitney test if both or one of the tests failed; otherwise, it was determined by Student’s *t* test. The efficiency of DRE was calculated using the following formula:

$$ \operatorname{RE}=\left(1\frac{{\operatorname{TD}}_{120}}{{\operatorname{TD}}_0}\right)\times 100\% $$

where TD_120_ is the percentage of DNA in the comet tail after 120 min repair incubation and TD_0_ is the percentage of DNA in the comet tail after exposure to hydrogen peroxide.

## Results

### Distribution of Genotypes of the Studied Polymorphisms

The distribution of SNP genotypes is shown in Table 2. The distribution of genotypes in all cases was in agreement with the Hardy–Weinberg equilibrium. Among the studied SNPs, only G/T heterozygote of c.-468T>G–*APEX1* was significantly associated with increased risk of rDD. In Supplementary Tables 1, 2, 3, 4, 5, 6, 7, and 8, we present additional analysis, where we divided the studied group into individuals with either higher or lower than median DRE, basal DNA damage recognized by the alkaline version of the comet assay, and oxidative DNA damage recognized by Nth or hOGG1.

**Table 2:** Distribution of genotypes and alleles of the studied single nucleotide polymorphism and the risk of recurrent depression disorder

Genotype/allele	*N* (frequency)		Crude OR (95% CI)	*p*
	Controls (59)	Depression (43)		
*NEIL1* c.*589G4C (rs4462560)				
C/C	39 (0.661)	34 (0.791)	1.937 (0.779–4.819)	0.155
C/G	19 (0.322)	7 (0.168)	0.409 (0.154–1.087)	0.073
G/G	1 (0.017)	2 (0.047)	2.829 (0.248–32.251)	0.402
C/G and G/G	20 (0.339)	9 (0.209)	0.516 (0.208–1.284)	0.155
*hOGG1* c.977C>G (rs1052133)				
C/C	39 (0.661)	31 (0.721)	1.325 (0.562–3.122)	0.520
C/G	16 (0.271)	12 (0.279)	1.040 (0.432–2.507)	0.930
G/G	4 (0.068)	0 (–)	–	–
C/G and G/G	20 (0.339)	12 (0.279)	0.755 (0.30–1.779)	0.520
*MUTYH* c.972G>C (rs3219489)				
C/C	40 (0.678)	26 (0.605)	0.726 (0.320–1.649)	0.445
C/G	17 (0.288)	13 (0.302)	1.071 (0.453–2.532)	0.877)
G/G	2 (0.034)	4 (0.093)	2.923 (0.510–16.747)	0.228
C/G and G/G	19 (0.322)	17 (0.395)	1.77 (0.606–3.124)	0.445
*PARP1* c.2285T>C (rs1136410)				
A/A	36 (0.610)	32 (0.744)	1.859 (0.785–4.401)	0.159
A/G	21 (0.356)	10 (0.233)	0.548 (0.226–1.330)	0.184
G/G	2 (0.034)	1 (0.023)	0.679 (0.060–7.733)	0.755
A/G and G/G	23 (0.390)	11 (0.256)	0.538 (0.227–1.274)	0.159
*XRCC1* c.1196A>G (rs25487)				
C/C	22 (0.373)	14 (0.326)	0.812 (0.355–1.858)	0.622
C/T	31 (0.525)	24 (0.558)	1.141 (0.518–2.513)	0.743
T/T	6 (0.102)	5 (0.116)	1.162 (0.330–4.089)	0.815
*XRCC1* c.580C>T (rs1799782)				
G/G	50 (0.847)	40 (0.930)	2.400 (0.609–9.456)	0.211
G/A	9 (0.153)	3 (0.070)	0.417 (0.106–1.642)	0.211
A/A	0 (–)	0 (–)	–	
*FEN1* c.-441G>A (rs174538)				
G/G	29 (0.492)	26 (0.605)	1.582 (0.713–3.508)	0.259
G/A	30 (0.508)	17 (0.395)	0.632 (0.285–1.402)	0.259
A/A	0 (–)	0 (–)	–	–
*APEX1* c.-468T>G (rs1760944)				
G/G	9 (0.153)	5 (0.116)	0.731 (0.226–2.359)	0.600
G/T	26 (0.441)	28 (0.651)	**2.369** (**1.053**–**5.330**)	**0.037**
T/T	24 (0.407)	10 (0.233)	0.442 (0.184–1.063)	0.068
*APEX1* c.444T>G (rs1130409)				
G/G	15 (0.254)	12 (0.279)	1.135 (0.468–2.758)	0.779
G/T	31 (0.525)	17 (0.395)	0.591 (0.266–1.310)	0.195
T/T	13 (0.220)	14 (0.326)	1.708 (0.704–4.145)	0.236
*LIG1* c.-7C>T (rs20579)				
G/G	48 (0.814)	33 (0.767)	0.756 (0.288–1.984)	0.570
G/A	9 (0.153)	10 (0.233)	1.684 (0.618–4.586)	0.308
A/A	2 (0.034)	0 (–)	–	–
G/A and A/A	11 (0.186))	10 (0.233)	1.322 (0.504–3.468)	0.570
*LIG3* c.*50C>T (rs1052536)				
C/C	14 (0.237)	8 (0.186)	0.735 (0.277–1.947)	0.535
C/T	27 (0.458)	20 (0.465)	1.031 (0.469–2.267)	0.940
T/T	18 (0.305)	15 (0.349)	1.220 (0.528–2.818)	0.641
*LIG3* c.*83A>C (rs4796030)				
A/A	9 (0.153)	5 (0.116)	0.731 (0.226–2.359)	0.600
A/C	26 (0.441)	18 (0.419)	0.914 (0.413–2.023)	0.824
C/C	24 (0.407)	20 (0.465)	1.268 (0.574–2.803)	0.557

*p* < 0.05 along with corresponding ORs are in bold

### Basal Endogenous DNA Damage

Table 3 presents the total basal DNA damage in depressed patients and controls, classified according to polymorphic variants. If there were less than three cases with either minor allele or heterozygote, these two groups were merged. We found that the patients with rDD had significantly higher endogenous DNA damage than the controls (*p* < 0.001). Moreover, we demonstrated that the level of DNA damage in each genotype of the studied polymorphisms was significantly higher in the depressed patients when compared to the controls (*p* < 0.05), but there was no difference between the genotypes within the groups (*p* > 0.05). Also, when we divided the population into those with higher and lower than median basal DNA damage, in each case, this damage was higher in the patients compared to the controls (Supplementary Tables 9 and 10). In a similar fashion to Table 3, Tables 4 and 5 present the mean oxidative DNA damage recognized by hOGG1 and Nth, respectively. In all cases, except for the heterozygote of c.580C>T–*XRCC1*, we found that depressed patients had higher oxidative DNA damage than those in the control group (*p* < 0.05) and that the differences between genotypes within these groups were not statistically significant (*p* > 0.05).

**Table 3:** Basal endogenous DNA damage

Genotype	Tail DNA (%), mean ± SEM		*p* ^a^
	Control	Depression	
Total			
–	1.86 ± 0.16	6.94 ± 0.55	**<0.001**
*NEIL1* c.*589G4C (rs4462560)			
C/C	1.74 ± 0.18	6.71 ± 0.64	**<0.001**
C/G and G/G	2.08 ± 0.29	7.78 ± 1.04	**<0.001**
*p* ^b^	0.337	0.310	
*hOGG1* c.977C>G (rs1052133)			
C/C	1.83 ± 0.20	6.73 ± 0.67	**<0.001**
C/G and G/G	1.90 ± 0.24	7.47 ± 0.96	**<0.001**
*p* ^b^	0.608	0.417	
*MUTYH* c.972G>C (rs3219489)			
C/C	1.75 ± 0.18	6.54 ± 0.54	**<0.001**
C/G and G/G	2.08 ± 0.29	7.54 ± 1.12	**<0.001**
*p* ^b^	0.368	0.619	
*PARP1* c.2285T>C (rs1136410)			
A/A	1.96 ± 0.21	7.15 ± 0.69	**<0.001**
A/G and G/G	1.69 ± 0.23	6.23 ± 0.83	**<0.001**
*p* ^b^	0.401	0.679	
*XRCC1* c.1196A>G (rs25487)			
C/C	1.50 ± 0.24	7.05 ± 0.84	**<0.001**
C/T	1.97 ± 0.20	6.65 ± 0.84	**<0.001**
T/T	2.55 ± 0.66	8.03 ± 1.03	**0.001**
*p* ^b^	0.084	0.304	
*XRCC1* c.580C>T (rs1799782)			
G/G	1.82 ± 0.17	6.87 ± 0.58	**<0.001**
G/A	2.04 ± 0.45	7.78 ± 1.46	**0.016**
*p* ^b^	0.635	0.404	
*FEN1* c.-441G>A (rs174538)			
G/G	1.80 ± 0.21	6.45 ± 0.41	**<0.001**
G/A	1.91 ± 0.24	7.67 ± 1.24	**<0.001**
*p* ^b^	0.940	0.960	
*APEX1* c.-468T>G (rs1760944)			
G/G	1.95 ± 0.25	7.36 ± 1.12	**<0.001**
G/T	1.71 ± 0.25	6.89 ± 0.72	**<0.001**
T/T	2.03 ± 0.27	6.34 ± 1.34	**0.008**
*p* ^b^	0.438	0.875	
*APEX1* c.444T>G (rs1130409)			
G/G	1.63 ± 0.25	8.59 ± 1.44	**<0.001**
G/T	1.81 ± 0.21	6.27 ± 0.77	**<0.001**
T/T	2.27 ± 0.41	6.33 ± 0.58	**<0.001**
*p* ^b^	0.545	0.242	
*LIG1* c.-7C>T (rs20579)			
G/G	1.89 ± 0.17	7.05 ± 0.68	**<0.001**
G/A and A/A	1.71 ± 0.38	6.55 ± 0.81	**<0.001**
*p* ^b^	0.559	1.000	
*LIG3* c.*50C>T (rs1052536)			
C/C	2.17 ± 0.40	5.22 ± 0.82	**0.001**
C/T	1.88 ± 0.20	7.68 ± 1.02	**<0.001**
T/T	1.58 ± 0.28	6.86 ± 0.60	**<0.001**
*p* ^b^	0.448	0.365	
*LIG3* c.*83A>C (rs4796030)			
A/A	1.77 ± 0.39	5.50 ± 0.77	**<0.001**
A/C	1.98 ± 0.21	7.27 ± 1.08	**<0.001**
C/C	1.75 ± 0.27	7.00 ± 0.65	**<0.001**
*p* ^b^	0.605	0.680	

^a^Values for patients vs. controls

^b^Values between different genotype carriers

*p* < 0.05 are in bold

**Table 4:** Oxidative DNA damage recognized by hOGG1

Genotype	Tail DNA (%), mean ± SEM		*p* ^a^
	Controls	Depression	
Total			
–	6.54 ± 0.22	14.88 ± 1.09	**<0.001**
*NEIL1* c.*589G4C (rs4462560)			
C/C	6.64 ± 0.30	14.63 ± 1.24	**<0.001**
C/G and G/G	6.36 ± 0.29	15.81 ± 2.42	**<0.001**
*p* ^b^	0.695	0.541	
*hOGG1* c.977C>G (rs1052133)			
C/C	6.56 ± 0.29	14.31 ± 1.27	**<0.001**
C/G and G/G	6.52 ± 0.32	16.34 ± 2.18	**<0.001**
*p* ^b^	0.994	0,394	
*MUTYH* c.972G>C (rs3219489)			
C/C	6.43 ± 0.23	15.27 ± 1.29	**<0.001**
C/G and G/G	6.77 ± 0.50	14.28 ± 1.99	**<0.001**
*p* ^b^	0.764	0.405	
*PARP1* c.2285T>C (rs1136410)			
A/A	6.37 ± 0.29	14.54 ± 1.22	**<0.001**
A/G and G/G	6.81 ± 0.33	15.87 ± 2.47	**<0.001**
*p* ^b^	0.273	0.568	
*XRCC1* c.1196A>G (rs25487)			
C/C	6.49 ± 0.33	14.67 ± 1.70	**<0.001**
C/T	6.60 ± 0.34	14.70 ± 1.62	**<0.001**
T/T	6.43 ± 0.53	16.21 ± 2.98	**0.004**
*p* ^b^	0.998	0.797	
*XRCC1* c.580C>T (rs1799782)			
G/G	6.40 ± 0.24	15.27 ± 1.14	**<0.001**
G/A	7.34 ± 0.51	12.37 ± 3.47	0.064
*p* ^b^	0.090	0.617	
*FEN1* c.-441G>A (rs174538)			
G/G	6.38 ± 0.33	13.34 ± 1.14	**<0.001**
G/A	6.70 ± 0.30	17.24 ± 2.07	**<0.001**
*p* ^b^	0.422	0.153	
*APEX1* c.-468T>G (rs1760944)			
G/G	6.70 ± 0.39	14.68 ± 2.38	**<0.001**
G/T	6.37 ± 0.27	14.66 ± 1.46	**<0.001**
T/T	6.61 ± 0.70	16.51 ± 1.11	**<0.001**
*p* ^b^	0.979	0.373	
*APEX1* c.444T>G (rs1130409)			
G/G	7.27 ± 0.48	17.66 ± 2.85	**<0.001**
G/T	6.33 ± 0.28	12.10 ± 1.48	**<0.001**
T/T	6.06 ± 0.41	15.87 ± 1.20	**<0.001**
*p* ^b^	0.126	0.076	
*LIG1* c.-7C>T (rs20579)			
G/G	6.56 ± 0.26	15.04 ± 1.31	**<0.001**
G/A and A/A	6.44 ± 0.35	15.14 ± 1.90	**0.001**
*p* ^b^	0.969	0.635	
*LIG3* c.*50C>T (rs1052536)			
C/C	5.97 ± 0.28	13.48 ± 1.20	**<0.001**
C/T	6.62 ± 0.34	14.89 ± 1.89	**<0.001**
T/T	6.87 ± 0.47	15.61 ± 1.84	**<0.001**
*p* ^b^	0.374	0.701	
*LIG3* c.*83A>C (rs4796030)			
A/A	6.45 ± 0.39	14.76 ± 1.35	**0.003**
A/C	6.30 ± 0.31	14.35 ± 2.07	**<0.001**
C/C	6.84 ± 0.41	15.38 ± 1.46	**<0.001**
*p* ^b^	0.789	0.423	

^a^Values for patients vs. controls

^b^Values between different genotype carriers

*p* < 0.05 are in bold

**Table 5:** Oxidative DNA damage recognized by Nth

Genotype	Tail DNA (%), mean ± SEM		*p* ^a^
	Controls	Depression	
Total			
–	5.81 ± 0.21	12.57 ± 0.877	**<0.001**
*NEIL1* c.*589G4C (rs4462560)			
C/C	5.85 ± 0.28	12.56 ± 1.03	**<0.001**
C/G and G/G	5.75 ± 0.32	12.64 ± 1.62	**<0.001**
*p* ^b^	0.642	0.823	
*hOGG1* c.977C>G (rs1052133)			
C/C	5.96 ± 0.30	12.16 ± 1.05	**<0.001**
C/G and G/G	5.53 ± 0.25	13.65 ± 1.60	**<0.001**
*p* ^b^	0.309	0.409	
*MUTYH* c.972G>C (rs3219489)			
C/C	5.84 ± 0.21	12.20 ± 0.91	**<0.001**
C/G and G/G	5.77 ± 0.51	13.15 ± 1.76	**<0.001**
*p* ^b^	0.390	0.891	
*PARP1* c.2285T>C (rs1136410)			
A/A	5.73 ± 0.31	12.25 ± 1.02	**<0.001**
A/G and G/G	5.93 ± 0.27	13.51 ± 1.75	**<0.001**
*p* ^b^	0.335	0.297	
*XRCC1* c.1196A>G (rs25487)			
C/C	5.71 ± 0.28	11.61 ± 0.99	**<0.001**
C/T	5.85 ± 0.35	12.73 ± 1.30	**<0.001**
T/T	6.01 ± 0.45	14.53 ± 3.49	**0.017**
*p* ^b^	0.914	0.827	
*XRCC1* c.580C>T (rs1799782)			
G/G	5.77 ± 0.24	12.78 ± 0,93	**<0.001**
G/A	6.08 ± 0.48	10.34 ± 1.50	**0.004**
*p* ^b^	0.317	0.489	
*FEN1* c.-441G>A (rs174538)			
G/G	5.83 ± 0.35	11.38 ± 0.80	**<0.001**
G/A	5.80 ± 0.25	14.40 ± 1.80	**<0.001**
*p* ^b^	0.693	0.391	
*APEX1* c.-468T>G (rs1760944)			
G/G	5.76 ± 0.39	13.00 ± 1.78	**<0.001**
G/T	5.98 ± 0.26	12.86 ± 1.16	**<0.001**
T/T	5.48 ± 0.59	10.12 ± 1.71	**0.008**
*p* ^b^	0.523	0.539	
*APEX1* c.444T>G (rs1130409)			
G/G	6.22 ± 0.55	14.98 ± 2.14	**<0.001**
G/T	5.62 ± 0.28	11.23 ± 1.29	**<0.001**
T/T	5.73 ± 0.22	12.15 ± 1.13	**<0.001**
*p* ^b^	0.522	0.279	
*LIG1* c.-7C>T (rs20579)			
G/G	5.89 ± 0.25	12.74 ± 1.09	**<0.001**
G/A and A/A	5.50 ± 0.34	12.17 ± 1.23	**<0.001**
*p* ^b^	0.330	0.656	
*LIG3* c.*50C>T (rs1052536)			
C/C	5.89 ± 0.39	10.07 ± 1.03	**<0.001**
C/T	5.98 ± 0.25	13.36 ± 1.54	**<0.001**
T/T	5.50 ± 0.52	12.87 ± 1.33	**<0.001**
*p* ^b^	0.220	0.365	
*LIG3* c.*83A>C (rs4796030)			
A/A	5.97 ± 0.42	11.32 ± 1.23	**<0.001**
A/C	5.94 ± 0.28	12.90 ± 1.70	**<0.001**
C/C	5.63 ± 0.41	12.59 ± 1.10	**<0.0** **01**
*p* ^b^	0.233	0.721	

^a^Values for patients vs. controls

^b^Values between different genotype carriers

*p* < 0.05 are in bold

### Efficiency of DNA Damage Repair

**Fig. 1:**
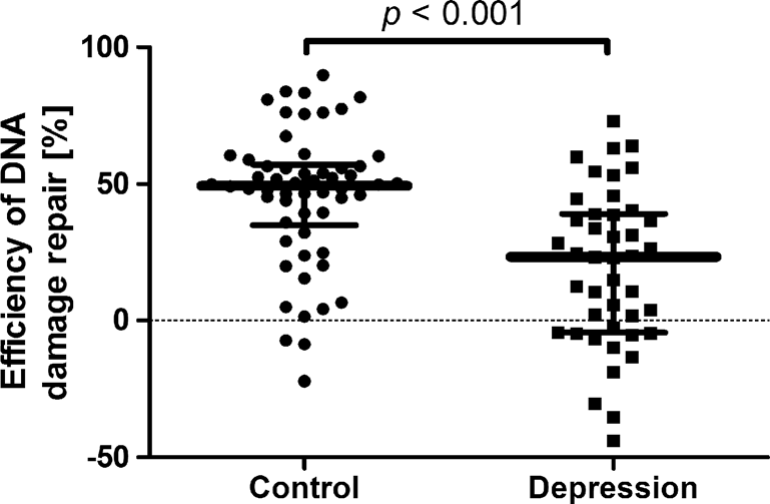
Efficiency of DNA damage repair in peripheral blood mononuclear cells of patients with depression and the control group. *Horizontal lines* denote the median, while *whiskers* show the interquartile range

In the overall studied population, DRE was significantly lower in the cases when compared to the controls (*p* < 0.001; Table 6 and Fig. 1). However, we did not find any statistically significant difference between the patients and controls in individuals with the following genotypes: combination of C/G and G/G of c.977C>G–*hOGG1*, combination of C/G and G/G of c.972G>C–*MUTYH*, combination of A/G and G/G of c.2285T>C–*PARP1*, T/T of c.1196A>G–*XRCC1*, G/A of c.580C>T–*XRCC1*, T/T of c.-468T>G–*APEX1*, T/T of c.444T>G–*APEX1*, and C/C and T/T of c.*50C>T–*LIG3* (Table 6). Moreover, within the groups of cases and controls, no statistically significant difference was found between the different genotype carriers (Table 6). Moreover, in individuals with rDD and with DRE higher than the median, we found differences between carriers of the genotypes of c.977C>G–*hOGG1* and c.972G>C–*MUTYH* (Table 7), while in the patients with lower than the median DRE, such differences were present between carriers of the genotypes of c.2285T>C–*PARP1* and c.-7C>T–*LIG1* (Table 8).

**Table 6:** Efficiency of DNA damage repair

Genotype	DRE (%), mean ± SEM		*p* ^a^
	Controls	Depression	
Total			
–	43.39 ± 3.91	19.38 ± 4.31	**<0.001**
*NEIL1* c.*589G4C (rs4462560)			
C/C	39.16 ± 5.29	15.71 ± 4.91	**<0.001**
C/G and G/G	51.63 ± 4.82	33.26 ± 7.83	**0.049**
*p* ^b^	0.333	0.098	
*hOGG1* c.977C>G (rs1052133)			
C/C	41.82 ± 4.95	17.94 ± 4.49	**<0.001**
C/G and G/G	46.44 ± 6.46	23.09 ± 10.54	0.054
*p* ^b^	0.476	0.599	
*MUTYH* c.972G>C (rs3219489)			
C/C	42.80 ± 4.97	15.12 ± 4.84	**<0.001**
C/G and G/G	44.62 ± 6.34	25.33 ± 7.95	0.064
*p* ^b^	0.801	0.251	
*PARP1* c.2285T>C (rs1136410)			
A/A	44.19 ± 5.31	17.34 ± 4.95	**<0.001**
A/G and G/G	42.14 ± 5.76	24.42 ± 8.93	0.066
*p* ^b^	0.858	0.480	
*XRCC1* c.1196A>G (rs25487)			
C/C	42.38 ± 5.31	15.87 ± 7.60	**0.004**
C/T	44.46 ± 5.76	15.75 ± 5.74	**<0.001**
T/T	41.55 ± 5.76	44.69 ± 7.41	0.840
*p* ^b^	0.797	0.097	
*XRCC1* c.580C>T (rs1799782)			
G/G	45.05 ± 4.24	19.76 ± 4.16	**<0.001**
G/A	34.18 ± 10.19	11.03 ± 32.20	0.370
*p* ^b^	0.143	0.617	
*FEN1* c.-441G>A (rs174538)			
G/G	38.13 ± 4.28	19.99 ± 4.97	**0.008**
G/A	48.48 ± 6.43	17.87 ± 8.00	**0.001**
*p* ^b^	0.065	0.813	
*APEX1* c.-468T>G (rs1760944)			
G/G	42.26 ± 7.47	9.83 ± 7.67	**0.001**
G/T	45.50 ± 4.25	21.35 ± 5.42	**0.003**
T/T	40.31 ± 11.55	25.48 ± 15.49	0.458
*p* ^b^	0.935	0.480	
*APEX1* c.444T>G (rs1130409)			
G/G	43.74 ± 6.50	11.90 ± 9.61	**0.008**
G/T	45.86 ± 4.42	16.00 ± 6.07	**<0.001**
T/T	38.41 ± 12.53	29.20 ± 7.06	0.152
*p* ^b^	0.949	0.255	
*LIG1* c.-7C>T (rs20579)			
G/G	40.37 ± 4.56	18.08 ± 5.15	**<0.001**
G/A and A/A	56.56 ± 5.37	22.68 ± 7.71	**0.002**
*p* ^b^	0.158	0.675	
*LIG3* c.*50C>T (rs1052536)			
C/C	42.44 ± 6.92	18.20 ± 9.66	**0.052**
C/T	49.02 ± 6.69	19.52 ± 6.24	**<0.001**
T/T	35.31 ± 6.37	19.17 ± 8.01	0.121
*p* ^b^	0.100	0.994	
*LIG3* c.*83A>C (rs4796030)			
A/A	51.64 ± 8.28	20.51 ± 11.63	**0.047**
A/C	46.10 ± 4.22	18.04 ± 6.82	**<0.001**
C/C	37.37 ± 7.87	19.81 ± 6.59	**0.022**
*p* ^b^	0.541	0.976	

^a^Values for patients vs. controls

^b^Values between different genotype carriers

*p* < 0.05 are in bold

**Table 7:** Efficiency of DNA damage repair (DRE) of persons with higher than the median DRE

Genotype	DRE (%), mean ± SEM		*p* ^a^
	Controls	Depression	
Total			
–	62.56 ± 2.34	42.21 ± 3.13	**<0.001**
*NEIL1* c.*589G4C (rs4462560)			
C/C	60.37 ± 2.82	41.98 ± 3.77	**0.004**
C/G and G/G	66.93 ± 4.03	42.70 ± 6.06	**0.003**
*p* ^b^	0.210	0.918	
*hOGG1* c.977C>G (rs1052133)			
C/C	61.20 ± 2.87	38.29 ± 3.01	**<0.001**
C/G and G/G	64.90 ± 4.10	52.67 ± 6.94	0.125
*p* ^b^	0.414	**0.037**	
*MUTYH* c.972G>C (rs3219489)			
C/C	61.40 ± 2.65	35.32 ± 2.60	**<0.001**
C/G and G/G	62.24 ± 4.90	52.04 ± 5.22	0.086
*p* ^b^	0.619	**0.005**	
*PARP1* c.2285T>C (rs1136410)			
A/A	65.35 ± 3.03	45.87 ± 4.04	**<0.001**
A/G and G/G	58.91 ± 3.54	36.93 ± 4.64	**0.005**
*p* ^b^	0.155	0.165	
*XRCC1* c.1196A>G (rs25487)			
C/C	61.88 ± 3.92	42.55 ± 6.93	**0.009**
C/T	63.17 ± 3.28	40.90 ± 4.20	**<0.001**
T/T	63.05 ± 6.92	44.69 ± 7.41	0.149
*p* ^b^	0.836	0.899	
*XRCC1* c.580C>T (rs1799782)			
G/G	63.02 ± 2.47	40.75 ± 2.90	**<0.001**
G/A	58.39 ± 8.59	72.96 ± 0.00	–
*p* ^b^	0.213	–	
*FEN1* c.-441G>A (rs174538)			
G/G	57.66 ± 2.63	39.61 ± 3.62	**0.006**
G/A	65.82 ± 3.32	46.77 ± 5.80	**0.006**
*p* ^b^	0.244	0.282	
*APEX1* c.-468T>G (rs1760944)			
G/G	66.20 ± 3.80	33.88 ± 2.44	**0.005**
G/T	58.42 ± 2.93	43.40 ± 3.71	**0.003**
T/T	67.60 ± 8.65	47.41 ± 14.04	0.252
*p* ^b^	0.195	0.576	
*APEX1* c.444T>G (rs1130409)			
G/G	60.28 ± 3.74	43.58 ± 6.43	**0.032**
G/T	64.08 ± 4.08	38.67 ± 4.40	**0.002**
T/T	62.65 ± 4.41	44.60 ± 5.84	**0.029**
*p* ^b^	0.928	0.709	
*LIG1* c.-7C>T (rs20579)			
G/G	61.57 ± 2.58	41.24 ± 3.37	**<0.001**
G/A and A/A	65.79 ± 5.61	46.61 ± 8.93	0.087
*p* ^b^	0.556	0.521	
*LIG3* c.*50C>T (rs1052536)			
C/C	60.12 ± 4.71	42.46 ± 6.37	0.051
C/T	64.53 ± 3.37	45.31 ± 5.15	**0.036**
T/T	59.81 ± 4.47	39.01 ± 5.20	**0.016**
*p* ^b^	0.420	0.682	
*LIG3* c.*83A>C (rs4796030)			
A/A	62.54 ± 4.91	38.36 ± 6.91	**0.025**
A/C	63.36 ± 3.82	45.70 ± 5.26	**0.012**
C/C	61.76 ± 3.99	40.72 ± 4.79	**0.007**
*p* ^b^	0.946	0.701	

^a^Values for patients vs. controls

^b^Values between different genotype carriers

*p* < 0.05 are in bold

**Table 8:** Efficiency of DNA damage repair (DRE) of persons with lower than the median DRE

Genotype	DRE (%), mean ± SEM		*p* ^a^
	Controls	Depression	
Total			
–	23.56 ± 5.57	−4.54 ± 3.66	**<0.001**
*NEIL1* c.*589G4C (rs4462560)			
C/C	16.84 ± 7.65	−5.04 ± 4.02	**0.002**
C/G and G/G	33.56 ± 5.69	0.22 ± 5.50	0.114
*p* ^b^	0.156	0.684	
*hOGG1* c.977C>G (rs1052133)			
C/C	23.42 ± 7.17	−3.76 ± 3.69	**<0.001**
C/G and G/G	23.88 ± 8.87	−6.49 ± 9.54	**0.041**
*p* ^b^	0.981	0.754	
*MUTYH* c.972G>C (rs3219489)			
C/C	22.25 ± 7.71	−5.08 ± 4.79	**0.002**
C/G and G/G	26.06 ± 7.28	−3.65 ± 6.03	**0.008**
*p* ^b^	0.731	0.855	
*PARP1* c.2285T>C (rs1136410)			
A/A	25.26 ± 7.38	−2.18 ± 3.39	**<0.001**
A/G and G/G	20.33 ± 7.28	−26.96 ± 17.00	**0.044**
*p* ^b^	0.477	0.043	
*XRCC1* c.1196A>G (rs25487)			
C/C	8.26 ± 16.62	−4.14 ± 5.34	0.279
C/T and T/T	29.39 ± 8.44	−4.78 ± 5.08	**<0.001**
*p* ^b^	0.213	0.934	
*XRCC1* c.580C>T (rs1799782)			
G/G	23.95 ± 6.41	−2.92 ± 3.68	**<0.001**
G/A	22.47 ± 12.01	−19.93 ± 15.29	0.121
*p* ^b^	0.809	0.179	
*FEN1* c.-441G>A (rs174538)			
G/G	24.34 ± 4.76	−2.08 ± 4.41	**<0.001**
G/A	22.47 ± 12.01	−7.82 ± 6.33	**0.017**
*p* ^b^	0.580	0.451	
*APEX1* c.-468T>G (rs1760944)			
G/G	22.01 ± 10.62	−4.58 ± 8.15	**0.025**
G/T	24.82 ± 5.61	−4.52 ± 4.16	**<0.001**
T/T	18.49 ± 12.95	−7.40 ± 2.56	0.286
*p* ^b^	0.905	0.974	
*APEX1* c.444T>G (rs1130409)			
G/G	25.13 ± 9.67	−10.73 ± 8.12	**0.029**
G/T	30.26 ± 4.65	−3.07 ± 4.91	**0.001**
T/T	1.48 ± 5.70	−0.38 ± 23.64	0.310
*p* ^b^	0.329	0.709	
*LIG1* c.-7C>T (rs20579)			
G/G	20.87 ± 6.28	−9.05 ± 4.35	**<0.001**
G/A and A/A	40.41 ± 4.22	6.74 ± 4.38	**<0.001**
*p* ^b^	0.242	**0.048**	
*LIG3* c.*50C>T (rs1052536)			
C/C	24.76 ± 9.02	−6.05 ± 1.57	**0.042**
C/T	23.33 ± 13	−0.69 ± 4.78	**0.003**
T/T	23.06 ± 6.98	−10.59 ± 9.46	**0.028**
*p* ^b^	0.717	0.522	
*LIG3* c.*83A>C (rs4796030)			
A/A	29.84 ± 18.58	−6.26 ± 3.70	0.232
A/C	31.04 ± 4.33	−3.63 ± 4.16	**<0.001**
C/C	12.97 ± 11.61	−5.75 ± 6.78	0.082
*p* ^b^	0.230	0.951	

^a^Values for patients vs. controls

^b^Values between different genotype carriers

*p* < 0.05 are in bold

## Discussion

A growing body of evidence suggests that elevated ROS and peroxidation of lipids both indicating oxidative stress, together with activated immune-inflammatory pathways, are present in depressed patients and may play an important role in the pathogenesis of this mental disorder [6, 8]. Moreover, in the serum, urine, and PBMCs of patients suffering from clinical symptoms of depression, elevated levels of 8-oxoG were found, showing that oxidative stress may compromise genetic material [9–14]. Our recent studies also demonstrated, via the comet assay, the significantly higher level of oxidatively modified purines and pyrimidines in PBMCs from depressed patients when compared to non-depressed control subjects [16]. Additionally, we found increased levels of DNA lesions, such as alkali labile sites and DNA strand breaks, in the PBMCs isolated from depressed patients. The consequent question is whether the increased DNA damage is caused by oxidative stress only or whether there are other contributing factors. The results of Yi et al. obtained in patients with milder forms of depression suggest the latter hypothesis because the levels of urinary 8-oxoG in these patients were not significantly different from those of the controls, suggesting that the amount of oxidative DNA damage may be dependent on the severity of the disease [15]. On the other hand, our previous results showed that the PBMCs of depressed patients repaired H_2_O_2_-induced DNA damage slower than those of the controls, suggesting an oxidative stress-independent mechanism [16]. One of the factors that may cause defective DNA damage repair is the presence of specific polymorphic variants of genes encoding proteins involved in this repair [18, 19]. Therefore, we genotyped 12 SNPs of BER genes in a larger group consisting of more than 550 participants and found that some of them can modulate rDD risk [17].

In the current work, we confirmed our previous results concerning DNA damage and repair on a slightly larger group than previously [16]. We found significantly greater amount of alkali labile sites and strand breaks recognized by the alkaline version of the comet assay in the PBMCs of cases when compared to the controls (Table 3). Similarly, the level of oxidized purines and pyrimidines, recognized by hOGG1 and Nth, respectively, was higher in patients than in controls (Tables 4 and 5). These results are consistent with those of other authors [9–14]. In the previous paper, we did not calculate DRE and, therefore, cannot compare this parameter between the present and our previous study [16]. However, the kinetics of DNA damage repair was monitored and showed that the cells from patients recovered more slowly than those of the controls, suggesting that DNA damage was less efficiently repaired in patients. In the present study, the DRE values were significantly lower in cases than in controls (Table 6), which is in agreement with our previous results and suggests impairments in the mechanisms of genetic material maintenance.

Additionally, we genotyped 12 SNPs located within either the coding or regulatory regions of genes involved in BER and found that only the heterozygote of c.-468T>G–*APEX1* significantly increased the risk of depression (*p* = 0.037; Table 2). This is consistent with our results (unpublished data) when the genotyping was done on a larger group of 599 depressed participants. Furthermore, other teams found that this polymorphism modulates promoter strength and the risk of lung cancer [30, 31]. We also observed that c.-468T>G–*APEX1* modulated the risk of rDD in subjects with higher basal DNA damage or lower oxidative DNA damage recognized by either Nth or hOGG1 (Supplementary Tables 3, 6, and 8). Moreover, the heterozygote of c.*589G>C–*NEIL* decreased the risk of depression in cases with lower DRE as well as lower oxidative DNA damage recognized by either Nth or hOGG1 (Supplementary Tables 2, 6, and 8), while the C/C homozygote increased this risk in patients with lower oxidative DNA damage recognized by Nth (Supplementary Table 6). Interestingly, in a previous paper, we also found that the homozygotes of this polymorphism modulated the risk of depression in a larger population of 555 participants [17]. This could indicate that c.-468T>G–*APEX1* and c.*589G>C–*NEIL* are strongly associated with depression.

We also tested whether the presence of the studied polymorphisms affected endogenous DNA damage and DRE. We divided participants according to the genotypes that they were carrying, and in all cases, endogenous basal DNA damage was higher in patients than in controls. Moreover, in case of comparison between genotype carriers within both groups, no difference in DNA damage was noted (Table 3). The same results were obtained for basal oxidative DNA damage recognized by either Nth or hOGG1, with the exception of the heterozygote of c.580C>T–*XRCC1* where the difference between the cases and controls was not statistically significant (Tables 4 and 5). These results—showing independence between the level of DNA damage and the SNP genotypes—may indicate that the polymorphisms did not affect DNA damage or that this effect was hidden by the damage caused by the disease itself. Either way, this argues in favor of the hypothesis that the elevated DNA damage found in depressed patients is mainly caused by the oxidative stress associated with this disease. On the other hand, our results obtained for DRE show that some polymorphic variants may affect DNA damage repair. DRE did not vary between the controls and the patients carrying the C/G and G/G of c.977C>G–*hOGG1*, the C/G and G/G of c.972G>C–*MUTYH*, the A/G and G/G of c.2285T>C–*PARP1*, the T/T of c.580C>T–*XRCC1*, the G/A of c.1196A>G–*XRCC1*, the T/T of c.444T>G–*APEX1*, the T/T of c.-468T>G–*APEX1*, or the C/C and T/T of c.*50C>T–*LIG3* (Table 6). It must be noted that, in all of these cases, the results did not differ between genotype carriers in either patients or controls. However, when we considered only cases with higher than the median DRE values, we found that this parameter was significantly greater in depressed patients with the C/G and G/G genotypes of either c.977C>G–*hOGG1* or c.972G>C–*MUTYH* (Table 7). Additionally, in patients with lower DRE, this parameter was higher in carriers of either the A/A of c.2285T>C–*PARP1* or G/A and A/A of c.-7C>T–*LIG1* (Table 8). Thus, these results show that the DNA lesions in depression may also originate from insufficient DNA damage repair and are at least partly caused by the presence of specific BER gene polymorphic variants.

The results should be discussed with regard to the strengths and limitations of our study. Firstly, a larger study sample would allow examining the associations with clinical characteristics, including age at onset of depression and number of episodes. On the other hand, similar studies had comparable number of participants [32, 33]. Secondly, we used PBMCs while examination of central nervous system (CNS) cells would give more information on the central aspects of the disease. Nevertheless, blood and CNS cells are both exposed to oxidative and nitrosative stressors [34, 35], and therefore blood cells are a good model to reflect what may happen in the CNS. The genetic constitution of PBMCs may reveal inherited defects in the constitution of other systems, including the CNS. Lastly, we genotyped only some SNPs while there are more polymorphisms that could possibly contribute to low DRE in depressed patients.

## Conclusion

This study shows that depression is accompanied by increased oxidative stress-induced DNA damage combined with an impaired DNA damage repair efficiency. Furthermore, the latter is in part related to specific SNPs of genes encoding proteins involved in BER. This pathway may play a role in the pathogenesis of depression and is likely a new drug target. Further studies are needed to develop and examine new drugs targeting oxidative stress, DNA damage, and DNA repair mechanisms.

## Electronic Supplementary Material

Below is the link to the electronic supplementary material.

Supplementary Table 1 Distribution of genotypes of the studied single-nucleotide polymorphism in the individuals with recurrent depression disorder and the controls with higher than median DRE (DOCX 19 kb)

Supplementary Table 2 Distribution of genotypes of the studied single-nucleotide polymorphism in the individuals with recurrent depression disorder and the controls with lower than median DRE (DOCX 20 kb)

Supplementary Table 3 Distribution of genotypes of the studied single-nucleotide polymorphism in the individuals with recurrent depression disorder and the controls with higher than median basal DNA damage (DOCX 19 kb)

Supplementary Table 4 Distribution of genotypes of the studied single-nucleotide polymorphism in the individuals with recurrent depression disorder and the controls with lower than median basal DNA damage (DOCX 19 kb)

Supplementary Table 5 Distribution of genotypes of the studied single-nucleotide polymorphism in the individuals with recurrent depression disorder and the controls with higher than median basal oxidative DNA damage recognized by Nth (DOCX 19 kb)

Supplementary Table 6 Distribution of genotypes of the studied single-nucleotide polymorphism in the individuals with recurrent depression disorder and the controls with lower than median basal oxidative DNA damage recognized by Nth (DOCX 20 kb)

Supplementary Table 7 Distribution of genotypes of the studied single-nucleotide polymorphism in the individuals with recurrent depression disorder and the controls with higher than median basal oxidative DNA damage recognized by hOGG1 (DOCX 19 kb)

Supplementary Table 8 Distribution of genotypes of the studied single-nucleotide polymorphism in the individuals with recurrent depression disorder and the controls with lower than median basal oxidative DNA damage recognized by hOGG1 (DOCX 20 kb)

Supplementary Table 9 Endogenous basal DNA damage higher than median (DOCX 18 kb)

Supplementary Table 10 Endogenous basal DNA damage lower than median (DOCX 18 kb)
