# Supplementary material for: Impact of Single Nucleotide Polymorphisms of Base Excision Repair Genes on DNA Damage and Efficiency of DNA Repair in Recurrent Depression Disorder
Source: Mol Neurobiol. 2016 Jun 21;54(6):4150–9. doi: 10.1007/s12035-016-9971-6 (PMC5509815; doi:10.1007/s12035-016-9971-6)
Supplement: Supplementary file 1 — Distribution of genotypes of the studied single-nucleotide polymorphism in the individuals with recurrent depression disorder and the controls with higher than median DRE (DOCX 19 kb) [file 12035_2016_9971_MOESM1_ESM.docx]

Supplementary Table 1. Distribution of genotypes of the studied single-nucleotide polymorphism in the individuals with recurrent depression disorder and the controls with higher than median DRE.

| Genotype/  allele | Controls  (30) | Depression  (22) | Crude OR (95% CI) | *p* |
| --- | --- | --- | --- | --- |
|  | N (Freq.) | N (Freq.) |  |  |
| *NEIL1* c.*589G4C (rs4462560) | | | | |
| C/C | 20 (0.667) | 15 (0.682) | 1.071 (0.331-3.470) | 0.908 |
| C/G | 10 (0.333) | 6 (0.273) | 0.750 (0.224-2.507) | 0.640 |
| G/G | 0 (-) | 1 (0.045) | - | - |
| C/G and G/G | 10 (0.333) | 7 (0.318) | 0.933 (0.288-3.023) | 0.908 |
| *hOGG1* c.977C>G (rs1052133) | | | | |
| C/C | 19 (0.633) | 16 (0.727) | 1.544 (0.467-5.108) | 0.477 |
| C/G | 10 (0.333) | 6 (0.273) | 0.750 (0.224-2.507) | 0.640 |
| G/G | 1 (0.033) | 0 (-) | - | - |
| C/G and G/G | 11 (0.367) | 6 (0.273) | 0.648 (0.196-2.143) | 0.477 |
| *MUTYH* c.972G>C (rs3219489) | | | | |
| C/C | 21 (0.700) | 13 (0.591) | 0.619 (0.195-1.963) | 0.415 |
| C/G | 8 (0.267) | 7 (0.318) | 1.283 (0.383-4.296) | 0.686 |
| G/G | 1 (0.033) | 2 (0.091) | 2.900 (0.246-34.187) | 0.398 |
| C/G and G/G | 9 (0.300) | 9 (0.409) | 1.615 (0.509-5.123) | 0.415 |
| *PARP1* c.2285T>C (rs1136410) | | | | |
| A/A | 17 (0.567) | 13 (0.591) | 1.105 (0.362-3.369) | 0.861 |
| A/G | 12 (0.400) | 8 (0.364) | 0.857 (0.276-2.667) | 0.790 |
| G/G | 1 (0.033) | 1 (0.045) | 1.381 (0.082-23.357) | 0.823 |
| A/G and G/G | 13 (0.433) | 9 (0.409) | 0.905 (0.297-2.762) | 0.861 |
| *XRCC1* c.1196A>G (rs25487) | | | | |
| C/C | 14 (0.467) | 6 (0.273) | 0.429 (0.132-1.396) | 0.160 |
| C/T | 13 (0.433) | 11 (0.500) | 1.308 (0.433-3.946) | 0.634 |
| T/T | 3 (0.100) | 5 (0.045) | 2.647 (0.559-12.530) | 0.220 |
| *XRCC1* c.580C>T (rs1799782) | | | | |
| G/G | 27 (0.900) | 21 (0.955) | 2.333 (0.226-24.076) | 0.477 |
| G/A | 3 (0.100) | 1 (0.045) | 0.429 (0.041-4.422) | 0.477 |
| A/A | 0 (-) | 0 (-) | - |  |
| *FEN1* c.-441G>A (rs174538) | | | | |
| G/G | 12 (0.400) | 14 (0.636) | 2.625 (0.844-8.166) | 0.096 |
| G/A | 18 (0.600) | 8 (0.045) | 0.381 (0.122-1.185) | 0.096 |
| A/A | 0 (-) | 0 (-) | - | - |
| *APEX1* c.-468T>G (rs1760944) | | | | |
| G/G | 11 (0.367) | 4 (0.182) | 1.026 (0.205-5.132) | 0.975 |
| G/T | 15 (0.500) | 15 (0.682) | 2.143 (0.680-6.752) | 0.193 |
| T/T | 4 (0.133) | 3 (0.136) | 0.384 (0.103-1.428) | 0.153 |
| *APEX1* c.444T>G (rs1130409) | | | | |
| G/G | 8 (0.267) | 5 (0.227) | 0.809 (0.224-2.921) | 0.746 |
| G/T | 14 (0.467) | 8 (0.364) | 0.653 (0.212-2.015) | 0.459 |
| T/T | 8 (0.267) | 9 (0.409) | 1.904 (0.589-6.156) | 0.282 |
| *LIG1* c.-7C>T (rs20579) | | | | |
| G/G | 23 (0.767) | 18 (0.818) | 1.370 (0.346-5.415) | 0.654 |
| G/A | 6 (0.200) | 4 (0.182) | 0.889 (0.218-3.623) | 0.869 |
| A/A | 1 (0.033) | 0 (-) | **-** | **-** |
| G/A and A/A | 7 (0.233) | 4 (0.182) | 0.730 (0.185-2.887) | 0.654 |
| *LIG3* c.*50C>T (rs1052536) | | | | |
| C/C | 7 (0.233) | 4 (0.182) | 0.730 (0.185-2.887) | 0.654 |
| C/T | 17 (0.567) | 9 (0.409) | 0529 (0.174-1.615) | 0.264 |
| T/T | 6 (0.200) | 9 (0.409) | 2.769 (0.806-9.512) | 0.106 |
| *LIG3* c.*83A>C (rs4796030) | | | | |
| A/A | 6 (0.200) | 3 (0.136) | 0.632 (0.139-2.862) | 0.551 |
| A/C | 12 (0.400) | 8 (0.364) | 0.857 (0.276-2.667) | 0.790 |
| C/C | 12 (0.400) | 11 (0.409) | 1.500 (0.494-4.551) | 0.474 |

*p* < 0.05 along with corresponding ORs are in bold
