# Supplementary material for: Impact of Single Nucleotide Polymorphisms of Base Excision Repair Genes on DNA Damage and Efficiency of DNA Repair in Recurrent Depression Disorder
Source: Mol Neurobiol. 2016 Jun 21;54(6):4150–9. doi: 10.1007/s12035-016-9971-6 (PMC5509815; doi:10.1007/s12035-016-9971-6)
Supplement: Supplementary file 2 — Distribution of genotypes of the studied single-nucleotide polymorphism in the individuals with recurrent depression disorder and the controls with lower than median DRE (DOCX 20 kb) [file 12035_2016_9971_MOESM2_ESM.docx]

Supplementary Table 2. Distribution of genotypes of the studied single-nucleotide polymorphism in the individuals with recurrent depression disorder and the controls with lower than median DRE.

| Genotype/  allele | Controls  (29) | Depression  (21) | Crude OR (95% CI) | *p* |
| --- | --- | --- | --- | --- |
|  | N (Freq.) | N (Freq.) |  |  |
| *NEIL1* c.*589G4C (rs4462560) | | | | |
| C/C | 19 (0.655) | 19 (0.905) | 5.000 (0.964-25.930) | 0.055 |
| C/G | 9 (0.310) | 1 (0.190) | **0.111 (0.013-0.961)** | **0.046** |
| G/G | 1 (0.034) | 1 (0.190) | 1.400 (0.083-23.737) | 0.816 |
| C/G and G/G | 10 (0.345) | 2 (0.095) | 0.200 (0.0386-1.037) | 0.055 |
| *hOGG1* c.977C>G (rs1052133) | | | | |
| C/C | 20 (0.690) | 15 (0.714) | 1.125 (0.329-3.853) | 0.851 |
| C/G | 6 (0.207) | 6 (0.286) | 1.533 (0.416-5.656) | 0.521 |
| G/G | 3 (0.103) | 0 (-) | - | - |
| C/G and G/G | 9 (0.310) | 6 (0.286) | 0.889 (0.260-3.044) | 0.851 |
| *MUTYH* c.972G>C (rs3219489) | | | | |
| C/C | 19 (0.655) | 13 (0.619) | 0.855 (0.266-2.748) | 0.793 |
| C/G | 9 (0.310) | 6 (0.286) | 0.889 (0.260-3.044) | 0.851 |
| G/G | 1 (0.034) | 2 (0.095) | 2.947 (0.249-34.850) | 0.391 |
| C/G and G/G | 10 (0.345) | 8 (0.381) | 1.169 (0.364-3.756) | 0.793 |
| *PARP1* c.2285T>C (rs1136410) | | | | |
| A/A | 19 (0.655) | 19 (0.905) | 5.000 (0.964-25.930) | 0.055 |
| A/G | 9 (0.310) | 2 (0.095) | 0.234 (0.045-1.225) | 0.086 |
| G/G | 1 (0.034) | 0 (-) | 0.679 (0.060-7.733) | 0.755 |
| A/G and G/G | 10 (0.345) | 2 (0.095) | 0.200 (0.039-1.037) | 0.055 |
| *XRCC1* c.1196A>G (rs25487) | | | | |
| C/C | 8 (0.276) | 8 (0.381) | 1.615 (0.487-5.361) | 0.433 |
| C/T | 18 (0.621) | 13 (0.619) | 0.993 (0.312-3.158) | 0.991 |
| T/T | 3 (0.103) | 0 (-) | - | - |
| T/T and C/T | 21 (0.724) | 13 (0.619) | 0.619 (0.187-2.054) | 0.433 |
| *XRCC1* c.580C>T (rs1799782) | | | | |
| G/G | 23 (0.793) | 19 (0.905) | 2.478 (0.447-13.727) | 0.299 |
| G/A | 6 (0.207) | 2 (0.095) | 0.404 (0.073-2.235) | 0.299 |
| A/A | 0 (-) | 0 (-) | - |  |
| *FEN1* c.-441G>A (rs174538) | | | | |
| G/G | 17 (0.586) | 12 (0.571) | 0.941 (0.302-2.934) | 0.917 |
| G/A | 12 (0.414) | 9 (0.429) | 1.062 (0.341-3.313) | 0.917 |
| A/A | 0 (-) | 0 (-) | - | - |
| *APEX1* c.-468T>G (rs1760944) | | | | |
| G/G | 13 (0.448) | 6 (0.286) | 0.505 (0.088-2.898) | 0.444 |
| G/T | 11 (0.379) | 13 (0.619) | 2.659 (0.836-8.456) | 0.098 |
| T/T | 5 (0.172) | 2 (0.095) | 0.492 (0.149-1.629) | 0.246 |
| G/T and T/T | 16 (0.552) | 15 (0.619) | 2.031 (0.614-6.721) | 0.246 |
| *APEX1* c.444T>G (rs1130409) | | | | |
| G/G | 7 (0.241) | 7 (0.333) | 1.571 (0.453-5.450) | 0.476 |
| G/T | 17 (0.586) | 9 (0.429) | 0.529 (0.170-1.651) | 0.273 |
| T/T | 5 (0.172) | 5 (0.238) | 1.500 (0.373-6.032) | 0.568 |
| *LIG1* c.-7C>T (rs20579) | | | | |
| G/G | 25 (0.862) | 15 (0.714) | 0.400 (0.097-1.651) | 0.205 |
| G/A | 3 (0.103) | 6 (0.286) | 3.467 (0.755-15.924) | 0.110 |
| A/A | 0 (-) | 1 (0.048) | **-** | **-** |
| G/A and A/A | 3 (0.103) | 7 (0.333) | 2.500 (0.606-10.321) | 0.205 |
| *LIG3* c.*50C>T (rs1052536) | | | | |
| C/C | 7 (0.241) | 4 (0.190) | 0.739 (0.410-2.945) | 0.669 |
| C/T | 10 (0.345) | 11 (0.524) | 2.090 (0.663-6.593) | 0.209 |
| T/T | 12 (0.414) | 6 (0.286) | 0.567 (0.171-1.883) | 0.354 |
| *LIG3* c.*83A>C (rs4796030) | | | | |
| A/A | 3 (0.103) | 2 (0.095) | 0.912 (0.139-6.005) | 0.924 |
| A/C | 14 (0.483) | 10 (0.476) | 0.974 (0.316-2.998) | 0.963 |
| C/C | 12 (0.414) | 9 (0.429) | 1.062 (0.341-3.313) | 0.917 |
| A/A and A/C | 17 (0.586) | 12 (0.571) | 0.941 (0.302-2.934) | 0.917 |

*p* < 0.05 along with corresponding ORs are in bold
