# Supplementary material for: Impact of Single Nucleotide Polymorphisms of Base Excision Repair Genes on DNA Damage and Efficiency of DNA Repair in Recurrent Depression Disorder
Source: Mol Neurobiol. 2016 Jun 21;54(6):4150–9. doi: 10.1007/s12035-016-9971-6 (PMC5509815; doi:10.1007/s12035-016-9971-6)
Supplement: Supplementary file 3 — Distribution of genotypes of the studied single-nucleotide polymorphism in the individuals with recurrent depression disorder and the controls with higher than median basal DNA damage (DOCX 19 kb) [file 12035_2016_9971_MOESM3_ESM.docx]

Supplementary Table 3. Distribution of genotypes of the studied single-nucleotide polymorphism in the individuals with recurrent depression disorder and the controls with higher than median basal DNA damage.

| Genotype/  allele | Controls  (30) | Depression  (22) | Crude OR (95% CI) | *p* |
| --- | --- | --- | --- | --- |
|  | N (Freq.) | N (Freq.) |  |  |
| *NEIL1* c.*589G4C (rs4462560) | | | | |
| C/C | 18 (0.621) | 16 (0.762) | 1.778 (0.541-5.838) | 0.343 |
| C/G | 11 (0.379) | 5 (0.238) | 0.508 (0.147-1.761) | 0.286 |
| G/G | 1 (0.034) | 1 (0.048) | 1.381 (0.082-23.357) | 0.823 |
| C/G and G/G | 12 (0.414) | 6 (0.286) | 0.563 (0.171-1.847) | 0.343 |
| *hOGG1* c.977C>G (rs1052133) | | | | |
| C/C | 19 (0.655) | 15 (0.714) | 1.241 (0.387-3.976) | 0.717 |
| C/G | 10 (0.345) | 7 (0.333) | 0.933 (0.288-3.023) | 0.908 |
| G/G | 1 (0.034) | 0 (-) | - | - |
| C/G and G/G | 11 (0.379) | 7 (0.333) | 0.806 (0.252-2.583) | 0.717 |
| *MUTYH* c.972G>C (rs3219489) | | | | |
| C/C | 18 (0.621) | 12 (0.571) | 0.800 (0.263-2.434) | 0.694 |
| C/G | 11 (0.379) | 9 (0.429) | 1.196 (0.387-3.697) | 0.756 |
| G/G | 1 (0.034) | 1 (0.048) | 1.381 (0.082-23.357) | 0.823 |
| C/G and G/G | 12 (0.414) | 10 (0.476) | 1.250 (0.411-3.803) | 0.694 |
| *PARP1* c.2285T>C (rs1136410) | | | | |
| A/A | 18 (0.621) | 17 (0.810) | 2.267 (0.659-7.802) | 0.194 |
| A/G | 10 (0.345) | 4 (0.190) | 0.444 (0.118-1.669) | 0.230 |
| G/G | 2 (0.069) | 1 (0.048) | 0.667 (0.057-7.852) | 0.747 |
| A/G and G/G | 12 (0.414) | 5 (0.238) | 0.441 (0.128-1.519) | 0.194 |
| *XRCC1* c.1196A>G (rs25487) | | | | |
| C/C | 8 (0.276) | 7 (0.333) | 1.283 (0.383-4.296) | 0.686 |
| C/T | 18 (0.621) | 11 (0.524) | 0.667 (0.220-2.023) | 0.474 |
| T/T | 4 (0.138) | 4 (0.238) | 1.444 (0.319-6.542) | 0.633 |
| *XRCC1* c.580C>T (rs1799782) | | | | |
| G/G | 25 (0.862) | 20 (0.952) | 2.000 (0.350-11.418) | 0.435 |
| G/A | 5 (0.172) | 2 (0.095) | 0.500 (0.088-2.855) | 0.435 |
| A/A | 0 (-) | 0 (-) | - |  |
| *FEN1* c.-441G>A (rs174538) | | | | |
| G/G | 14 (0.483) | 14 (0.667) | 2.000 (0.648-6.170) | 0.228 |
| G/A | 16 (0.552) | 8 (0.381) | 0.500 (0.162-1.542) | 0.228 |
| A/A | 0 (-) | 0 (-) | - | - |
| *APEX1* c.-468T>G (rs1760944) | | | | |
| G/G | 14 (0.483) | 6 (0.286) | 0.429 (0.132-1.396) | 0.160 |
| G/T | 10 (0.345) | 14 (0.667) | **3.500 (1.104-11.094)** | **0.033** |
| T/T | 6 (0.207) | 2 (0.095) | 0.400 (0.073-2.204) | 0.293 |
| G/T and T/T | 16 (0.552) | 16 (0.762) | 2.333 (0.716-7.601) | 0.160 |
| *APEX1* c.444T>G (rs1130409) | | | | |
| G/G | 6 (0.207) | 8 (0.381) | 2.286 (0.657-7.954) | 0.194 |
| G/T | 17 (0.586) | 8 (0.381) | 0.437 (0.141-1.352) | 0.151 |
| T/T | 7 (0.241) | 6 (0.286) | 1.232 (0.348-4.359) | 0.746 |
| *LIG1* c.-7C>T (rs20579) | | | | |
| G/G | 26 (0.897) | 16 (0.762) | 0.410 (0.100-1.681) | 0.216 |
| G/A | 3 (0.103) | 6 (0.286) | 3.375 (0.740-15.394) | 0.116 |
| A/A | 1 (0.034) | 0 (-) | **-** | **-** |
| A/A and G/A | 4 (0.138) | 6 (0.286) | 2.437 (0.595-9.985) | 0.216 |
| *LIG3* c.*50C>T (rs1052536) | | | | |
| C/C | 7 (0.241) | 2 (0.095) | 0.329 (0.061-1.766) | 0.195 |
| C/T | 16 (0.552) | 11 (0.524) | 0.875 (0.291-2.632) | 0.812 |
| T/T | 7 (0.241) | 9 (0.429) | 2.275 (0.686-7.546) | 0.179 |
| C/C and C/T | 14 (0.483) | 11 (0.524) | 0.440 (0.133-1.458) | 0.179 |
| *LIG3* c.*83A>C (rs4796030) | | | | |
| A/A | 5 (0.172) | 1 (0.048) | 0.238 (0.026-2.201) | 0.206 |
| A/C | 13 (0.448) | 9 (0.429) | 0.905 (0.297-2.762) | 0.861 |
| C/C | 12 (0.414) | 12 (0.571) | 1.800 (0.592-5.476) | 0.300 |
| A/A and A/C | 17 (0.586) | 13 (0.524) | 0.556 (0.183-1.690) | 0.300 |

*p* < 0.05 along with corresponding ORs are in bold
