# Supplementary material for: Impact of Single Nucleotide Polymorphisms of Base Excision Repair Genes on DNA Damage and Efficiency of DNA Repair in Recurrent Depression Disorder
Source: Mol Neurobiol. 2016 Jun 21;54(6):4150–9. doi: 10.1007/s12035-016-9971-6 (PMC5509815; doi:10.1007/s12035-016-9971-6)
Supplement: Supplementary file 4 — Distribution of genotypes of the studied single-nucleotide polymorphism in the individuals with recurrent depression disorder and the controls with lower than median basal DNA damage (DOCX 19 kb) [file 12035_2016_9971_MOESM4_ESM.docx]

Supplementary Table 4. Distribution of genotypes of the studied single-nucleotide polymorphism in the individuals with recurrent depression disorder and the controls with lower than median basal DNA damage.

| Genotype/  allele | Controls  (29) | Depression  (21) | Crude OR(95% CI) | *p* |
| --- | --- | --- | --- | --- |
|  | N (Freq.) | N (Freq.) |  |  |
| *NEIL1* c.*589G4C (rs4462560) | | | | |
| C/C | 21 (0724) | 18 (0.571) | 2.286 (0.526-9.928) | 0.270 |
| C/G | 8 (0.276) | 2 (0.095) | 0.276 (0.052-1.467) | 0.131 |
| G/G | 0 (-) | 1 (0.048) | - | - |
| C/G and G/G | 8 (0.276) | 3 (0.143) | 0.438 (0.101-1.900) | 0.270 |
| *hOGG1* c.977C>G (rs1052133) | | | | |
| C/C | 20 (0.690) | 16 (0.762) | 1.440 (0.402-5.157) | 0.575 |
| C/G | 6 (0.207) | 5 (0.238) | 1.198 (0.311-4.609) | 0.793 |
| G/G | 3 (0.103) | 0 (-) | - | - |
| C/G and G/G | 9 (0.310) | 5 (0.238) | 0.694 (0.194-2.487) | 0.575 |
| *MUTYH* c.972G>C (rs3219489) | | | | |
| C/C | 22 (0.759) | 14 (0.667) | 0.636 (0.183-2.207) | 0.476 |
| C/G | 6 (0.207) | 4 (0.190) | 0.902 (0.220-3.702) | 0.886 |
| G/G | 1 (0.034) | 3 (0.143) | 4.667 (0.450-48.414) | 0.197 |
| C/G and G/G | 7 (0.241) | 7 (0.333) | 1.571 (0.453-5.450) | 0.476 |
| *PARP1* c.2285T>C (rs1136410) | | | | |
| A/A | 18 (0.621) | 15 (0.714) | 1.528 (0457-5.113) | 0.492 |
| A/G | 11 (0.379) | 6 (0.190) | 0.655 (0.196-2.190) | 0.492 |
| G/G | 0 (-) | 0 (-) | - | - |
| *XRCC1* c.1196A>G (rs25487) | | | | |
| C/C | 14 (0.483) | 7 (0.333) | 0.536 (0.167-1.715) | 0.293 |
| C/T | 13 (0.448) | 13 (0.619) | 2.000 (0.636-6.286) | 0.235 |
| T/T | 2 (0.069) | 1 (0.048) | 0.675 (0.057-7.973) | 0.755 |
| T/T and C/T | 15 (0.517) | 14 (0.333) | 1.867 (0.583-5.975) | 0.293 |
| *XRCC1* c.580C>T (rs1799782) | | | | |
| G/G | 25 (0.862) | 20 (0.952) | 3.200 (0.331-30.938) | 0.315 |
| G/A | 4 (0.138) | 1 (0.048) | 0.313 (0.032-3.021) | 0.531 |
| A/A | 0 (-) | 0 (-) | - |  |
| *FEN1* c.-441G>A (rs174538) | | | | |
| G/G | 15 (0.517) | 12 (0.571) | 0.804 (0.260-2.488) | 0.705 |
| G/A | 14 (0.483) | 9 (0.429) | 1.244 (0.402-3.853) | 0.705 |
| A/A | 0 (-) | 0 (-) | - | - |
| *APEX1* c.-468T>G (rs1760944) | | | | |
| G/G | 10 (0.345) | 4 (0.190) | 0.447 (0.118-1.693) | 0.236 |
| G/T | 16 (0.552) | 14 (0.667) | 1.625 (0.507-5.213) | 0.414 |
| T/T | 3 (0.103) | 3 (0.143) | 1.444 (0.261-7.982) | 0.673 |
| *APEX1* c.444T>G (rs1130409) | | | | |
| G/G | 9 (0.310) | 4 (0.190) | 0523 (0.136-2.004) | 0.344 |
| G/T | 14 (0.483) | 9 (0.429) | 0.804 (0.260-2.488) | 0.705 |
| T/T | 6 (0.207) | 8 (0.381) | 2.359 (0.670-8.301) | 0.181 |
| *LIG1* c.-7C>T (rs20579) | | | | |
| G/G | 22 (0.759) | 17 (0.810) | 1.352 (0.340-5.386) | 0.669 |
| G/A | 6 (0.207) | 4 (0.190) | 0.902 (0.220-3.702) | 0.886 |
| A/A | 1 (0.034) | 0 (-) | **-** | **-** |
| A/A and G/A | 7 (0.241) | 4 (0.190) | 0.739 (0.186-2.945) | 0.669 |
| *LIG3* c.*50C>T (rs1052536) | | | | |
| C/C | 7 (0.241) | 6 (0.286) | 1.257 (0.352-4.489) | 0.725 |
| C/T | 11 (0.379) | 9 (0.429) | 1.227 (0.391-3.854) | 0.726 |
| T/T | 11 (0.379) | 6 (0.286) | 0.655 (0.196-2.190) | 0.492 |
| *LIG3* c.*83A>C (rs4796030) | | | | |
| A/A | 4 (0.138) | 4 (0.190) | 1.471 (0.323-6.702) | 0.618 |
| A/C | 13 (0.448) | 9 (0.429) | 0.923 (0.297-2.865) | 0.890 |
| C/C | 12 (0.414) | 8 (0.381) | 0.872 (0.276-2.752) | 0.815 |

*p* < 0.05 along with corresponding ORs are in bold
