# Supplementary material for: Impact of Single Nucleotide Polymorphisms of Base Excision Repair Genes on DNA Damage and Efficiency of DNA Repair in Recurrent Depression Disorder
Source: Mol Neurobiol. 2016 Jun 21;54(6):4150–9. doi: 10.1007/s12035-016-9971-6 (PMC5509815; doi:10.1007/s12035-016-9971-6)
Supplement: Supplementary file 5 — Distribution of genotypes of the studied single-nucleotide polymorphism in the individuals with recurrent depression disorder and the controls with higher than median basal oxidative DNA damage recognized by Nth (DOCX 19 kb) [file 12035_2016_9971_MOESM5_ESM.docx]

Supplementary Table 5. Distribution of genotypes of the studied single-nucleotide polymorphism in the individuals with recurrent depression disorder and the controls with higher than median basal oxidative DNA damage recognized by Nth.

| Genotype/  allele | Controls  (30) | Depression  (22) | Crude OR (95% CI) | *p* |
| --- | --- | --- | --- | --- |
|  | N (Freq.) | N (Freq.) |  |  |
| *NEIL1* c.*589G4C (rs4462560) | | | | |
| C/C | 22 (0.733) | 16 (0.727) | 0.970 (0.281-3.348) | 0.961 |
| C/G | 8 (0.267) | 6 (0.273) | 1.031 (0.299-3.560) | 0.961 |
| G/G | 0 (-) | 0 (-) | - | - |
| *hOGG1* c.977C>G (rs1052133) | | | | |
| C/C | 22 (0.733) | 16 (0.727) | 0.970 (0.281-3.348) | 0.961 |
| C/G | 6 (0.200) | 6 (0.273) | 1.500 (0.410-5.484) | 0.540 |
| G/G | 2 (0.067) | 0 (-) | - | - |
| C/G and G/G | 8 (0.267) | 6 (0.273) | 1.031 (0.299-3.560) | 0.961 |
| *MUTYH* c.972G>C (rs3219489) | | | | |
| C/C | 23 (0.767) | 13 (0.591) | 0.440 (0.133-1.458) | 0.179 |
| C/G | 7 (0.233) | 9 (0.409) | 2.275 (0.686-7.546) | 0.179 |
| G/G | 0 (-) | 0 (-) | - | - |
| *PARP1* c.2285T>C (rs1136410) | | | | |
| A/A | 15 (0.500) | 14 (0.636) | 1.750 (0.568-5.393) | 0.330 |
| A/G | 13 (0.433) | 7 (0.318) | 0.610 (0.193-1.931) | 0.401 |
| G/G | 2 (0.067) | 1 (0.045) | 0.667 (0.057-7.852) | 0.747 |
| A/G and G/G | 15 (0.500) | 8 (0.364) | 0.571 (0.185-1.761) | 0.330 |
| *XRCC1* c.1196A>G (rs25487) | | | | |
| C/C | 10 (0.333) | 6 (0.273) | 0.750 (0.224-2.507) | 0.640 |
| C/T | 16 (0.533) | 12 (0.545) | 1.050 (0.348-3.167) | 0.931 |
| T/T | 4 (0.133) | 4 (0.182) | 1.444 (0.319-6.542) | 0.633 |
| *XRCC1* c.580C>T (rs1799782) | | | | |
| G/G | 24 (0.800) | 21 (0.955) | 5.250 (0.584-47.217) | 0.139 |
| G/A | 6 (0.200) | 1 (0.045) | 0.190 (0.021-1.713) | 0.139 |
| A/A | 0 (-) | 0 (-) | - | - |
| *FEN1* c.-441G>A (rs174538) | | | | |
| G/G | 13 (0.433) | 13 (0.591) | 1.889 (0.619-5.762) | 0.264 |
| G/A | 17 (0.567) | 9 (0.045) | 0.529 (0.174-1.615) | 0.264 |
| A/A | 0 (-) | 0 (-) | - | - |
| *APEX1* c.-468T>G (rs1760944) | | | | |
| G/G | 11 (0.367) | 7 (0.318) | 0.806 (0.252-2.583) | 0.717 |
| G/T | 16 (0.533) | 13 (0.591) | 1.264 (0.416-3.843) | 0.680 |
| T/T | 3 (0.100) | 2 (0.091) | 0.900 (0.137-5.900) | 0.913 |
| G/T and T/T | 19 (0.633) | 15 (0.682) | 1.241 (0.387-3.976) | 0.717 |
| *APEX1* c.444T>G (rs1130409) | | | | |
| G/G | 9 (0.300) | 8 (0.364) | 1.333 (0.415-4.288) | 0.629 |
| G/T | 15 (0.500) | 6 (0.273) | 0.375 (0.115-1.220) | 0.103 |
| T/T | 6 (0.200) | 8 (0.364) | 2.286 (0.657-7.954) | 0.194 |
| *LIG1* c.-7C>T (rs20579) | | | | |
| G/G | 26 (0.867) | 15 (0.682) | 0.330 (0.083-1.314) | 0.116 |
| G/A | 4 (0.133) | 7 (0.318) | 3.033 (0.761-12.095) | 0.116 |
| A/A | 0 (-) | 0 (-) | **-** | **-** |
| *LIG3* c.*50C>T (rs1052536) | | | | |
| C/C | 8 (0.267) | 2 (0.091) | 0.275 (0.052-1.452) | 0.128 |
| C/T | 14 (0.467) | 11 (0.500) | 1.143 (0.380-3.438) | 0.812 |
| T/T | 8 (0.267) | 9 (0.409) | 1.904 (0.589-6.156) | 0.282 |
| C/C and C/T | 22 (0.733) | 13 (0.591) | 0.525 (0.162-1.698) | 0.282 |
| *LIG3* c.*83A>C (rs4796030) | | | | |
| A/A | 6 (0.200) | 2 (0.091) | 0.400 (0.073-2.204) | 0.293 |
| A/C | 14 (0.467) | 8 (0.364) | 0.653 (0.212-2.015) | 0.459 |
| C/C | 10 (0.333) | 12 (0.545) | 2.400 (0.774-7.441) | 0.129 |
| A/A and A/C | 20 (0.667) | 10 (0.455) | 0.417 (0.134-1.292) | 0.129 |

*p* < 0.05 along with corresponding ORs are in bold
