# Supplementary material for: Impact of Single Nucleotide Polymorphisms of Base Excision Repair Genes on DNA Damage and Efficiency of DNA Repair in Recurrent Depression Disorder
Source: Mol Neurobiol. 2016 Jun 21;54(6):4150–9. doi: 10.1007/s12035-016-9971-6 (PMC5509815; doi:10.1007/s12035-016-9971-6)
Supplement: Supplementary file 6 — Distribution of genotypes of the studied single-nucleotide polymorphism in the individuals with recurrent depression disorder and the controls with lower than median basal oxidative DNA damage recognized by Nth (DOCX 20 kb) [file 12035_2016_9971_MOESM6_ESM.docx]

Supplementary Table 6. Distribution of genotypes of the studied single-nucleotide polymorphism in the individuals with recurrent depression disorder and the controls with lower than median basal oxidative DNA damage recognized by Nth.

| Genotype/  allele | Controls  (29) | Depression  (21) | Crude OR (95% CI) | *p* |
| --- | --- | --- | --- | --- |
|  | N (Freq.) | N (Freq.) |  |  |
| *NEIL1* c.*589G4C (rs4462560) | | | | |
| C/C | 17 (0.586) | 18 (0.857) | **4.235 (1.015-17.668)** | **0.048** |
| C/G | 11 (0.379) | 1 (0.048) | **0.082 (0.010-0.698)** | **0.022** |
| G/G | 1 (0.034) | 2 (0.095) | 2.947 (0.249-34.850) | 0.391 |
| C/G and G/G | 12 (0.414) | 3 (0.143) | **0.236 (0.057-0.985)** | **0.048** |
| *hOGG1* c.977C>G (rs1052133) | | | | |
| C/C | 17 (0.586) | 15 (0.714) | 1.765 (0.531-5.865) | 0.354 |
| C/G | 10 (0.345) | 6 (0.286) | 0.760 (0.225-2.568) | 0.659 |
| G/G | 2 (0.069) | 0 (-) | - | - |
| C/G and G/G | 12 (0.414) | 6 (0.286) | 0.567 (0.171-1.883) | 0.354 |
| *MUTYH* c.972G>C (rs3219489) | | | | |
| C/C | 17 (0.586) | 13 (0.619) | 1.147 (0.363-3.621) | 0.815 |
| C/G | 10 (0.345) | 4 (0.190) | 0.447 (0.118-1.693) | 0.236 |
| G/G | 2 (0.069) | 4 (0.190) | 3.176 (0.524-19.266) | 0.209 |
| C/G and G/G | 12 (0.414) | 8 (0.381) | 0.872 (0.276-2.752) | 0.815 |
| *PARP1* c.2285T>C (rs1136410) | | | | |
| A/A | 21 (0.724) | 18 (0.857) | 2.286 (0.526-9.928) | 0.270 |
| A/G | 8 (0.276) | 3 (0.143) | 0.438 (0.101-1.900) | 0.270 |
| G/G | 0 (-) | 0 (-) | - | - |
| *XRCC1* c.1196A>G (rs25487) | | | | |
| C/C | 12 (0.414) | 8 (0.381) | 0.872 (0.276-2.752) | 0.815 |
| C/T | 15 (0.517) | 12 (0.571) | 1.244 (0.402-3.853) | 0.705 |
| T/T | 2 (0.069) | 1 (0.048) | 0.675 (0.057-7.973) | 0.755 |
| T/T and C/T | 17 (0.586) | 13 (0.619) | 1.147 (0.363-3.621) | 0.815 |
| *XRCC1* c.580C>T (rs1799782) | | | | |
| G/G | 26 (0.897) | 19 (0.905) | 1.096 (0.167-7.216) | 0.924 |
| G/A | 3 (0.103) | 2 (0.095) | 0.912 (0.139-6.005) | 0.924 |
| A/A | 0 (-) | 0 (-) | - |  |
| *FEN1* c.-441G>A (rs174538) | | | | |
| G/G | 16 (0.552) | 13 (0.619) | 1.320 (0.420-4.149) | 0.634 |
| G/A | 13 (0.448) | 8 (0.381) | 0.757 (0.241-2.380) | 0.634 |
| A/A | 0 (-) | 0 (-) | - | - |
| *APEX1* c.-468T>G (rs1760944) | | | | |
| G/G | 13 (0.448) | 3 (0.143) | **0.205 (0.049-0.853)** | **0.029** |
| G/T | 10 (0.345) | 15 (0.714) | **4.750 (1.406 -16.051)** | **0.012** |
| T/T | 6 (0.207) | 3 (0.143) | 0.639 (0.140-2.912) | 0.563 |
| G/T and T/T | 16 (0.552) | 18 (0.857) | **4.875 (1.173-20.261)** | **0.029** |
| *APEX1* c.444T>G (rs1130409) | | | | |
| G/G | 5 (0.172) | 4 (0.190) | 0.902 (0.220-3.702) | 0.886 |
| G/T | 16 (0.552) | 11 (0.524) | 0.894 (0.290-2.757) | 0.845 |
| T/T | 7 (0.241) | 6 (0.286) | 1.257 (0.352-4.489) | 0.725 |
| *LIG1* c.-7C>T (rs20579) | | | | |
| G/G | 22 (0.759) | 18 (0.857) | 1.909 (0.431-8.463) | 0.395 |
| G/A | 5 (0.172) | 3 (0.143) | 0.800 (0.169-3.793) | 0.779 |
| A/A | 2 (0.069) | 0 (-) | **-** | **-** |
| A/A and G/A | 7 (0.241) | 3 (0.143) | 0.524 (0.118-2.322) | 0.395 |
| *LIG3* c.*50C>T (rs1052536) | | | | |
| C/C | 6 (0.207) | 6 (0.286) | 1.533 (0.416-5.656) | 0.521 |
| C/T | 13 (0.448) | 9 (0.429) | 0.923 (0.297-2.865) | 0.890 |
| T/T | 10 (0.345) | 6 (0.286) | 0.760 (0.225-2.568) | 0.659 |
| *LIG3* c.*83A>C (rs4796030) | | | | |
| A/A | 3 (0.103) | 3 (0.143) | 1.444 (0.261-7.982) | 0.673 |
| A/C | 12 (0.414) | 10 (0.476) | 1.288 (0.416-3.991) | 0.661 |
| C/C | 14 (0.483) | 8 (0.381) | 0.659 (0.210-2.068) | 0.475 |

*p* < 0.05 along with corresponding ORs are in bold
