# Supplementary material for: Impact of Single Nucleotide Polymorphisms of Base Excision Repair Genes on DNA Damage and Efficiency of DNA Repair in Recurrent Depression Disorder
Source: Mol Neurobiol. 2016 Jun 21;54(6):4150–9. doi: 10.1007/s12035-016-9971-6 (PMC5509815; doi:10.1007/s12035-016-9971-6)
Supplement: Supplementary file 7 — Distribution of genotypes of the studied single-nucleotide polymorphism in the individuals with recurrent depression disorder and the controls with higher than median basal oxidative DNA damage recognized by hOGG1 (DOCX 19 kb) [file 12035_2016_9971_MOESM7_ESM.docx]

Supplementary Table 7. Distribution of genotypes of the studied single-nucleotide polymorphism in the individuals with recurrent depression disorder and the controls with higher than median basal oxidative DNA damage recognized by hOGG1.

| Genotype/  allele | Controls  (30) | Depression  (22) | Crude OR (95% CI) | *p* |
| --- | --- | --- | --- | --- |
|  | N (Freq.) | N (Freq.) |  |  |
| *NEIL1* c.*589G4C (rs4462560) | | | | |
| C/C | 21 (0.700) | 16 (0.727) | 1.143 (0.337-3.874) | 0.830 |
| C/G | 9 (0.300) | 6 (0.273) | 0.875 (0.258-2.966) | 0.830 |
| G/G | 0 (-) | 0 (-) | - | - |
| *hOGG1* c.977C>G (rs1052133) | | | | |
| C/C | 20 (0.667) | 15 (0.682) | 1.071 (0.331-3.470) | 0.908 |
| C/G | 7 (0.233) | 7 (0.318) | 1.533 (0.447-5.262) | 0.497 |
| G/G | 3 (0.100) | 0 (-) | - | - |
| C/G and G/G | 10 (0.333) | 7 (0.318) | 0.933 (0.288-3.023) | 0.908 |
| *MUTYH* c.972G>C (rs3219489) | | | | |
| C/C | 22 (0.733) | 15 (0.682) | 0.779 (0.233-2.608) | 0.686 |
| C/G | 7 (0.233) | 7 (0.318) | 1.533 (0.447-5.262) | 0.497 |
| G/G | 1 (0.033) | 0 (-) | - | - |
| C/G and G/G | 8 (0.267) | 7 (0.318) | 1.283 (0.383-4.296) | 0.686 |
| *PARP1* c.2285T>C (rs1136410) | | | | |
| A/A | 16 (0.533) | 15 (0.682) | 1.875 (0.595-5.914) | 0.283 |
| A/G | 13 (0.433) | 6 (0.273) | 0.490 (0.150-1.602) | 0.238 |
| G/G | 1 (0.033) | 1 (0.045) | 1.381 (0.082-23.357) | 0.823 |
| A/G and G/G | 14 (0.467) | 7 (0.318) | 0.533 (0.169-1.682) | 0.283 |
| *XRCC1* c.1196A>G (rs25487) | | | | |
| C/C | 11 (0.367) | 8 (0.364) | 0.987 (0.315-3.095) | 0.982 |
| C/T | 16 (0.533) | 11 (0.500) | 0.875 (0.291-2.632) | 0.812 |
| T/T | 3 (0.100) | 3 (0.136) | 1.421 (0.258-7.814) | 0.686 |
| *XRCC1* c.580C>T (rs1799782) | | | | |
| G/G | 23 (0.767) | 21 (0.955) | 6.391 (0.725-56.381) | 0.095 |
| G/A | 7 (0.233) | 1 (0.045) | 0.156 (0.018-1.380) | 0.095 |
| A/A | 0 (-) | 0 (-) | - | - |
| *FEN1* c.-441G>A (rs174538) | | | | |
| G/G | 13 (0.433) | 11 (0.500) | 1.889 (0.619-5.762) | 0.264 |
| G/A | 17 (0.567) | 11 (0.500) | 0.529 (0.174-1.615) | 0.264 |
| A/A | 0 (-) | 0 (-) | - | - |
| *APEX1* c.-468T>G (rs1760944) | | | | |
| G/G | 12 (0.400) | 5 (0.227) | 0.441 (0.128-1.519) | 0.194 |
| G/T | 14 (0.467) | 13 (0.591) | 1.651 (0.543-5.020) | 0.377 |
| T/T | 4 (0.133) | 4 (0.182) | 1.444 (0.319-6.542) | 0.633 |
| *APEX1* c.444T>G (rs1130409) | | | | |
| G/G | 11 (0.367) | 7 (0.318) | 0.806 (0.252-2.583) | 0.717 |
| G/T | 13 (0.433) | 7 (0.318) | 0.610 (0.193-1.931) | 0.401 |
| T/T | 6 (0.200) | 8 (0.364) | 2.286 (0.657-7.954) | 0.194 |
| *LIG1* c.-7C>T (rs20579) | | | | |
| G/G | 24 (0.800) | 16 (0.727) | 0.667 (0.182-2.437) | 0.540 |
| G/A | 5 (0.167) | 6 (0.273) | 1.875 (0.490-7.179) | 0.359 |
| A/A | 1 (0.033) | 0 (-) | **-** | **-** |
| G/A and A/A | 6 (0.200) | 6 (0.273) | 1.500 (0.410-5.484) | 0.540 |
| *LIG3* c.*50C>T (rs1052536) | | | | |
| C/C | 5 (0.167) | 3 (0.136) | 0.789 (0.167-3.722) | 0.765 |
| C/T | 15 (0.500) | 9 (0.409) | 0.692 (0.228-2.103) | 0.517 |
| T/T | 10 (0.333) | 10 (0.455) | 1.667 (0.538-5.168) | 0.376 |
| *LIG3* c.*83A>C (rs4796030) | | | | |
| A/A | 6 (0.200) | 3 (0.136) | 0.632 (0.139-2.862) | 0.551 |
| A/C | 12 (0.400) | 6 (0.273) | 0.563 (0.171-1.847) | 0.343 |
| C/C | 12 (0.400) | 13 (0.591) | 2.167 (0.706-6.645) | 0.176 |

*p* < 0.05 along with corresponding ORs are in bold
