# Supplementary material for: Impact of Single Nucleotide Polymorphisms of Base Excision Repair Genes on DNA Damage and Efficiency of DNA Repair in Recurrent Depression Disorder
Source: Mol Neurobiol. 2016 Jun 21;54(6):4150–9. doi: 10.1007/s12035-016-9971-6 (PMC5509815; doi:10.1007/s12035-016-9971-6)
Supplement: Supplementary file 8 — Distribution of genotypes of the studied single-nucleotide polymorphism in the individuals with recurrent depression disorder and the controls with lower than median basal oxidative DNA damage recognized by hOGG1 (DOCX 20 kb) [file 12035_2016_9971_MOESM8_ESM.docx]

Supplementary Table 8. Distribution of genotypes of the studied single-nucleotide polymorphism in the individuals with recurrent depression disorder and the controls with lower than median basal oxidative DNA damage recognized by hOGG1.

| Genotype/  allele | Controls  (29) | Depression  (21) | Crude OR (95% CI) | *p* |
| --- | --- | --- | --- | --- |
|  | N (Freq.) | N (Freq.) |  |  |
| *NEIL1* c.*589G4C (rs4462560) | | | | |
| C/C | 18 (0.621) | 18 (0.857) | 3.667 (0.874-15.384) | 0.076 |
| C/G | 10 (0.345) | 1 (0.048) | **0.095 (0.011-0.815)** | **0.032** |
| G/G | 1 (0.034) | 2 (0.095) | 2.947 (0.249-34.850) | 0.391 |
| C/G and G/G | 11 (0.379) | 3 (0.143) | 0.273 (0.065-1.144) | 0.076 |
| *hOGG1* c.977C>G (rs1052133) | | | | |
| C/C | 19 (0.655) | 16 (0.762) | 1.684 (0.476-5.954) | 0.418 |
| C/G | 9 (0.310) | 5 (0.238) | 0.694 (0.194-2.487) | 0.575 |
| G/G | 1 (0.034) | 0 (-) | - | - |
| C/G and G/G | 10 (0.345) | 5 (0.238) | 0.594 (0.168-2.099) | 0.418 |
| *MUTYH* c.972G>C (rs3219489) | | | | |
| C/C | 18 (0.621) | 11 (0.524) | 0.672 (0.215-2.099) | 0.494 |
| C/G | 10 (0.345) | 6 (0.286) | 0.760 (0.225-2.568) | 0.659 |
| G/G | 1 (0.034) | 4 (0.190) | 6.588 (0.679-63.940) | 0.104 |
| C/G and G/G | 11 (0.379) | 10 (0.476) | 1.488 (0.477-4.644) | 0.494 |
| *PARP1* c.2285T>C (rs1136410) | | | | |
| A/A | 20 (0.690) | 17 (0.810) | 1.912 (0.499-7.330) | 0.344 |
| A/G | 8 (0.276) | 4 (0.190) | 0.618 (0.159-2.406) | 0.487 |
| G/G | 1 (0.034) | 0 (-) | - | - |
| A/G and G/G | 9 (0.310) | 4 (0.190) | 0.523 (0.136-2.004) | 0.344 |
| *XRCC1* c.1196A>G (rs25487) | | | | |
| C/C | 11 (0.379) | 6 (0.286) | 0.655 (0.196-2.190) | 0.492 |
| C/T | 15 (0.517) | 13 (0.619) | 1.517 (0.484-4.756) | 0.475 |
| T/T | 3 (0.103) | 2 (0.095) | 0.912 (0.139-6.005) | 0.924 |
| T/T and C/T | 18 (0.621) | 15 (0.714) | 1.528 (0.457-5.113) | 0.492 |
| *XRCC1* c.580C>T (rs1799782) | | | | |
| G/G | 27 (0.931) | 19 (0.905) | 0.704 (0.091-5.444) | 0.736 |
| G/A | 2 (0.069) | 2 (0.095) | 1.421 (0.184-10.994) | 0.736 |
| A/A | 0 (-) | 0 (-) | - |  |
| *FEN1* c.-441G>A (rs174538) | | | | |
| G/G | 16 (0.552) | 15 (0.714) | 1.308 (0.433-3.946) | 0.634 |
| G/A | 13 (0.448) | 6 (0.286) | 0.765 (0.253-2.308) | 0.634 |
| A/A | 0 (-) | 0 (-) | - | - |
| *APEX1* c.-468T>G (rs1760944) | | | | |
| G/G | 12 (0.414) | 5 (0.238) | 0.443 (0.127-1.540) | 0.200 |
| G/T | 12 (0.414) | 15 (0.714) | **3.542 (1.066-11.771)** | **0.039** |
| T/T | 5 (0.172) | 1 (0.048) | 0.240 (0.026-2.226) | 0.209 |
| G/T and T/T | 17 (0.586) | 16 (0.762) | 2.259 (0.649-7.859) | 0.200 |
| *APEX1* c.444T>G (rs1130409) | | | | |
| G/G | 4 (0.138) | 5 (0.238) | 1.953 (0.455-8.384) | 0.368 |
| G/T | 18 (0.621) | 10 (0.476) | 0.556 (0.178-1.734) | 0.312 |
| T/T | 7 (0.241) | 6 (0.286) | 1.257 (0.352-4.489) | 0.725 |
| *LIG1* c.-7C>T (rs20579) | | | | |
| G/G | 24 (0.828) | 17 (0.810) | 1.471 (0.323-6.702) | 0.618 |
| G/A | 4 (0.138) | 4 (0.190) | 0.885 (0.207-3.791) | 0.870 |
| A/A | 1 (0.034) | 0 (-) | **-** | **-** |
| A/A and G/A | 5 (0.172) | 4 (0.190) | 1.129 (0.264-4.835) | 0.870 |
| *LIG3* c.*50C>T (rs1052536) | | | | |
| C/C | 9 (0.310) | 5 (0.238) | 0.694 (0.194-2.487) | 0.575 |
| C/T | 12 (0.414) | 11 (0.524) | 1.558 (0.503-4.830) | 0.442 |
| T/T | 8 (0.276) | 5 (0.238) | 0.820 (0.225-2.989) | 0.764 |
| *LIG3* c.*83A>C (rs4796030) | | | | |
| A/A | 3 (0.103) | 2 (0.095) | 0.912 (0.139-6.005) | 0.924 |
| A/C | 14 (0.483) | 12 (0.571) | 1.429 (0.461-4.423) | 0.536 |
| C/C | 12 (0.414) | 7 (0.333) | 0.708 (0.220-2.283) | 0.564 |
| A/A and A/C | 17 (0.586) | 14 (0.667) | 1.412 (0.438-4.549) | 0.564 |

*p* < 0.05 along with corresponding ORs are in bold
