# Supplementary material for: Impact of Single Nucleotide Polymorphisms of Base Excision Repair Genes on DNA Damage and Efficiency of DNA Repair in Recurrent Depression Disorder
Source: Mol Neurobiol. 2016 Jun 21;54(6):4150–9. doi: 10.1007/s12035-016-9971-6 (PMC5509815; doi:10.1007/s12035-016-9971-6)
Supplement: Supplementary file 9 — Endogenous basal DNA damage higher than median (DOCX 18 kb) [file 12035_2016_9971_MOESM9_ESM.docx]

Supplementary Table 9. Endogenous basal DNA damage higher than median

| Genotype | Controls  Tail DNA (%)  Mean ± SEM | Depression  Tail DNA (%)  Mean ± SEM | *p*^*^ |
| --- | --- | --- | --- |
| Total | | | |
| - | 2.79 ± 0.17 | 9.34 ± 0.75 | **< 0.001** |
| *NEIL1* c.*589G4C (rs4462560) | | | |
| C/C | 2.77 ± 0.19 | 9.37 ± 0.96 | **< 0.001** |
| C/G and G/G | 2.81 ± 0.33 | 9.24 ± 1.14 | **< 0.001** |
| *p*^#^ | 0.767 | 0.971 |  |
| *hOGG1* c.977C>G (rs1052133) | | | |
| C/C | 2.85 ± 0.25 | 9.21 ± 1.02 | **< 0.001** |
| C/G and G/G | 2.68 ± 0.21 | 9.60 ± 1.00 | **< 0.001** |
| *p*^#^ | 0.983 | 0.438 |  |
| *MUTYH* c.972G>C (rs3219489) | | | |
| C/C | 2.81 ± 0.20 | 8.88 ± 0.63 | **< 0.001** |
| C/G and G/G | 2.76 ± 0.32 | 8.28 ± 1.50 | **< 0.001** |
| *p*^#^ | 0.596 | 0.869 |  |
| *PARP1* c.2285T>C (rs1136410) | | | |
| A/A | 2.97 ± 0.22 | 9.56 ± 0.95 | **< 0.001** |
| A/G and G/G | 2.51 ± 0.27 | 8.59 ± 0.70 | **0.002** |
| *p*^#^ | 0.099 | 1.000 |  |
| *XRCC1* c.1196A>G (rs25487) | | | |
| C/C | 2.69 ± 0.37 | 9.47 ± 0.96 | **< 0.001** |
| C/T | 2.69 ± 0.21 | 9.44 ± 1.39 | **< 0.001** |
| T/T | 3.42 ± 0.54 | 8.83 ± 0.84 | **0.002** |
| *p*^#^ | 0.243 | 0.788 |  |
| *XRCC1* c.580C>T (rs1799782) | | | |
| G/G | 1.82 ± 0.17 | 6.87 ± 0.58 | **< 0.001** |
| G/A | 2.04 ± 0.45 | 7.78 ± 1.46 | **0.002** |
| *p*^#^ | 0.504 | 0.775 |  |
| *FEN1* c.-441G>A (rs174538) | | | |
| G/G | 2.68 ± 0.25 | 8.13 ± 0.24 | **< 0.001** |
| G/A | 2.88 ± 0.24 | 11.44 ± 1.87 | **< 0.001** |
| *p*^#^ | 0.506 | 0.232 |  |
| *APEX1* c.-468T>G (rs1760944) | | | |
| G/G | 2.80 ± 0.25 | 9.50 ± 1.12 | **< 0.001** |
| G/T | 3.02 ± 0.25 | 9.29 ± 0.72 | **< 0.001** |
| T/T | 2.36 ± 0.27 | 9.20 ± 1.34 | **0.008** |
| *p*^#^ | 0.317 | 0.658 |  |
| *APEX1* c.444T>G (rs1130409) | | | |
| G/G | 2.64 ± 0.41 | 10.41 ± 1.86 | **< 0.001** |
| G/T | 2.57 ± 0.21 | 8.91 ± 0.93 | **< 0.001** |
| T/T | 3.44 ± 0.35 | 8.47 ± 0.42 | **< 0.001** |
| *p*^#^ | 0.107 | 0.704 |  |
| *LIG1* c.-7C>T (rs20579) | | | |
| G/G | 2.76 ± 0.17 | 9.73 ± 1.01 | **< 0.001** |
| G/A and A/A | 2.96 ± 0.71 | 8.28 ± 0.56 | **< 0.001** |
| *p*^#^ | 1.000 | 0.685 |  |
| *LIG3* c.*50C>T (rs1052536) | | | |
| C/C | 3.41 ± 0.39 | 8.45 ± 0.34 | **< 0.001** |
| C/T | 2.53 ± 0.19 | 10.30 ± 1.42 | **< 0.001** |
| T/T | 2.74 ± 0.40 | 8.36 ± 0.50 | **< 0.001** |
| *p*^#^ | 0.074 | 0.575 |  |
| *LIG3* c.*83A>C (rs4796030) | | | |
| A/A and A/C | 2.79 ± 0.19 | 9.95 ± 1.50 | **< 0.001** |
| C/C | 2.78 ± 0.33 | 8.82 ± 0.63 | **< 0.001** |
| *p*^#^ | 0.611 | 0.621 |  |

*p*^*^ – patients vs controls

*p*^#^ – between different genotypes carriers
