# Supplementary material for: Impact of Single Nucleotide Polymorphisms of Base Excision Repair Genes on DNA Damage and Efficiency of DNA Repair in Recurrent Depression Disorder
Source: Mol Neurobiol. 2016 Jun 21;54(6):4150–9. doi: 10.1007/s12035-016-9971-6 (PMC5509815; doi:10.1007/s12035-016-9971-6)
Supplement: Supplementary file 10 — Endogenous basal DNA damage lower than median (DOCX 18 kb) [file 12035_2016_9971_MOESM10_ESM.docx]

Supplementary Table 10. Endogenous basal DNA damage lower than median

| Genotype | Controls  Tail DNA (%)  Mean ± SEM | Depression  Tail DNA (%)  Mean ± SEM | *p*^*^ |
| --- | --- | --- | --- |
| Total | | | |
| - | 0.89 ± 0.08 | 4.42 ± 0.23 | **< 0.001** |
| *NEIL1* c.*589G4C (rs4462560) | | | |
| C/C | 0.86 ± 0.09 | 4.35 ± 0.26 | **< 0.001** |
| C/G and G/G | 0.99 ± 0.15 | 4.84 ± 0.38 | **< 0.001** |
| *p*^#^ | 0.436 | 0.481 |  |
| *hOGG1* c.977C>G (rs1052133) | | | |
| C/C | 0.87 ± 0.08 | 4.40 ± 0.28 | **< 0.001** |
| C/G and G/G | 0.95 ± 0.17 | 4.47 ± 0.46 | **0.003** |
| *p*^#^ | 0.621 | 1.000 |  |
| *MUTYH* c.972G>C (rs3219489) | | | |
| C/C | 0.88 ± 0.09 | 4.54 ± 0.29 | **< 0.001** |
| C/G and G/G | 0.92 ± 0.16 | 4.19 ± 0.41 | **< 0.001** |
| *p*^#^ | 0.845 | 0.455 |  |
| *PARP1* c.2285T>C (rs1136410) | | | |
| A/A | 0.95 ± 0.10 | 4.41 ± 0.28 | **< 0.001** |
| A/G and G/G | 0.80 ± 0.10 | 4.44 ± 0.45 | **0.001** |
| *p*^#^ | 0.333 | 1.000 |  |
| *XRCC1* c.1196A>G (rs25487) | | | |
| C/C | 0.83 ± 0.11 | 4.62 ± 0.41 | **< 0.001** |
| C/T and T/T | 0.95 ± 0.11 | 4,32 ± 0.29 | **< 0.001** |
| *p*^#^ | 0.437 | 0.332 |  |
| *XRCC1* c.580C>T (rs1799782) | | | |
| G/G | 0.90 ± 0.08 | 4.38 ± 0.24 | **< 0.001** |
| G/A | 0.87 ± 0.25 | 5.21 ± 0 | **-** |
| *p*^#^ | 0.922 | - |  |
| *FEN1* c.-441G>A (rs174538) | | | |
| G/G | 0.99 ± 0.10 | 4.49 ± 0.32 | **< 0.001** |
| G/A | 0.79 ± 0.11 | 4.32 ± 0.36 | **< 0.001** |
| *p*^#^ | 0.114 | 0.728 |  |
| *APEX1* c.-468T>G (rs1760944) | | | |
| G/G | 0.76 ± 0.12 | 4.15 ± 0.54 | **< 0.001** |
| G/T | 0.89 ± 0.10 | 4.50 ± 0.26 | **< 0.001** |
| T/T | 1.35 ± 0.15 | 4.43 ± 1.02 | **0.041** |
| *p*^#^ | 0.086 | 0.691 |  |
| *APEX1* c.444T>G (rs1130409) | | | |
| G/G | 0.95 ± 0.14 | 4.93 ± 1.44 | **< 0.001** |
| G/T | 0.85 ± 0.11 | 3.93 ± 0.36 | **< 0.001** |
| T/T | 0.90 ± 0.17 | 4.72 ± 0.39 | **< 0.001** |
| *p*^#^ | 0.842 | 0.181 |  |
| *LIG1* c.-7C>T (rs20579) | | | |
| G/G | 0.86 ± 0.09 | 4.53 ± 0.25 | **< 0.001** |
| G/A and A/A | 1.00 ± 0.12 | 3.94 ± 0.63 | **< 0.001** |
| *p*^#^ | 0.427 | 0.336 |  |
| *LIG3* c.*50C>T (rs1052536) | | | |
| C/C | 0.93 ± 0.20 | 4.14 ± 0.56 | **0.001** |
| C/T | 0.92 ± 0.13 | 4.48 ± 0.31 | **< 0.001** |
| T/T | 0.84 ± 0.09 | 4.61 ± 0.45 | **0.001** |
| *p*^#^ | 0.879 | 0.751 |  |
| *LIG3* c.*83A>C (rs4796030) | | | |
| A/A | 0.63 ± 0.12 | 4.84 ± 0.51 | **0.029** |
| A/C | 1.14± 0.10 | 4.38 ± 0.36 | **< 0.001** |
| C/C | 0.72 ± 0.11 | 4.26 ± 0.42 | **< 0.001** |
| *p*^#^ | **0.009** | 0.534 |  |

*p*^*^ – patients vs controls

*p*^#^ – between different genotypes carriers
